# System Pharmacology-Based Dissection of the Synergistic Mechanism of Huangqi and Huanglian for Diabetes Mellitus

**DOI:** 10.3389/fphar.2017.00694

**Published:** 2017-10-05

**Authors:** Shi-Jun Yue, Juan Liu, Wu-Wen Feng, Fei-Long Zhang, Jian-Xin Chen, Lan-Ting Xin, Cheng Peng, Hua-Shi Guan, Chang-Yun Wang, Dan Yan

**Affiliations:** ^1^Beijing Shijitan Hospital, Capital Medical University, Beijing, China; ^2^Key Laboratory of Marine Drugs (Ministry of Education of China), School of Medicine and Pharmacy, Ocean University of China, Qingdao, China; ^3^Laboratory for Marine Drugs and Bioproducts, Qingdao National Laboratory for Marine Science and Technology, Qingdao, China; ^4^College of Pharmacy, Chengdu University of Traditional Chinese Medicine, Chengdu, China; ^5^Information Center, Beijing University of Chinese Medicine, Beijing, China

**Keywords:** Huangqi, Huanglian, synergistic mechanism, diabetes, system pharmacology

## Abstract

The rapidly increasing diabetes mellitus (DM) is becoming a major global public health issue. Traditional Chinese medicine (TCM) has a long history of the treatment of DM with good efficacy. Huangqi and Huanglian are one of the most frequently prescribed herbs for DM, and the combination of them occurs frequently in antidiabetic formulae. However, the synergistic mechanism of Huangqi (*Radix Astragali*) and Huanglian (*Rhizoma Coptidis*) has not been clearly elucidated. To address this problem, a feasible system pharmacology model based on chemical, pharmacokinetic and pharmacological data was developed via network construction approach to clarify the synergistic mechanisms of these two herbs. Forty-three active ingredients of Huangqi (mainly astragalosides and isoflavonoids) and Huanglian (primarily isoquinoline alkaloids) possessing favorable pharmacokinetic profiles and biological activities were selected, interacting with 50 DM-related targets to provide potential synergistic therapeutic actions. Systematic analysis of the constructed networks revealed that these targets such as GLUT2, NOS2, PTP1B, and IGF1R were mainly involved in PI3K-Akt signaling pathway, insulin resistance, insulin signaling pathway, and HIF-1 signaling pathway, and were mainly located in retina, pancreatic islet, smooth muscle, immunity-related organ tissues, and whole blood. The contribution index of every active ingredient also indicated five compounds, including berberine (BBR), astragaloside IV (AIV), quercetin, palmatine, and astragalus polysaccharides, as the principal components of this herb combination. These results successfully explained the polypharmcological and synergistic mechanisms underlying the efficiency of Huangqi and Huanglian for the treatment of DM and its complications.

## Introduction

Diabetes mellitus (DM) is a chronic metabolic disorder influenced by interactions between genetic and environmental factors. The global prevalence of DM among adults aged 20–79 years was 8.8% in 2015, and its incidence is increasing rapidly (International Diabetes Federation, 2015). As complementary and alternative medicine, traditional Chinese medicine (TCM) has been proven to possess satisfactory effectiveness toward DM and its complications clinically, such as Gegen Qinlian Decoction (Tong et al., 2011) and Huangqi San (Xu et al., 2015), wherein Huangqi (*Radix Astragali*, the dried roots of *Astragalus membranaceus* (Fisch.) Bunge. var. *mongholicus* (Bunge.) Hsiao or *A. membranaceus* (Fisch.) Bunge, Fabaceae) and Huanglian (*Rhizoma Coptidis*, the rhizomes of *Coptis chinensis* Franch, Ranunculaceae) are one of the most frequently prescribed herbs (Xie et al., 2011). Specifically, Huangqi is widely used in East Asia to reinforce *Qi* (Ma et al., 2002) and its use to treat DM, classified as Xiao Ke syndrome in TCM, has been firstly documented in *Shen Nong Ben Cao Jing* (206 BC–24 AD, Western Han Dynasty). Huangqi has been developed into the intravenous injection (mainly astragalosides) in China to treat DM with good clinical effects (Nie et al., 2014). As a holy herb to treat Xiao Ke syndrome, Huanglian is frequently used in diabetic care partially due to its antihyperglycemic, antihyperlipidemic, antihypertensive, anti-inflammatory, and antioxidant activities (Tong et al., 2011; Pang et al., 2015). Based on a previous statistics, Huanglian has been used as a major ingredient in many antidiabetic Chinese patent medicines (CPMs) approved by the China Food and Drug Administration and the majority of them are combined with Huangqi, such as Jinqi Jiangtang tablets, Xiaokeping tablets, Tangmaikang capsules, and Shenjing Zhike Wan (Xie et al., 2011). However, although the combination of Huangqi and Huanglian has been frequently used in antidiabetic formulae and CPMs (Supplementary Figure S1), we still know little about how the active ingredients in Huangqi and Huanglian modulate the synergistic network for combating DM.

System pharmacology is emerging as a holistic and efficient tool to study the role of TCM due to its capable of describing complex interactions between drugs and biological systems including the human body, organs, and diseases from a network perspective (Kloft et al., 2016; Zhang et al., 2016). Combined with pharmacology and pharmacodynamics, it has been successfully applied to interpret the synergistic mechanisms of herb combinations at molecular network level (Zhou et al., 2016; Yu et al., 2017; Yue et al., 2017). In the present study, we tried to establish the compound-target (C-T), target-pathway (T-P), and target-organ (T-O) networks by the system pharmacology model based on chemical, pharmacokinetic and pharmacological data at the system, organ, and molecular levels (Supplementary Figure S2), so as to uncover the underlying synergistic mechanisms of Huangqi and Huanglian for treating DM.

## Materials and methods

### Chemical ingredients database building

**Figure 1:**
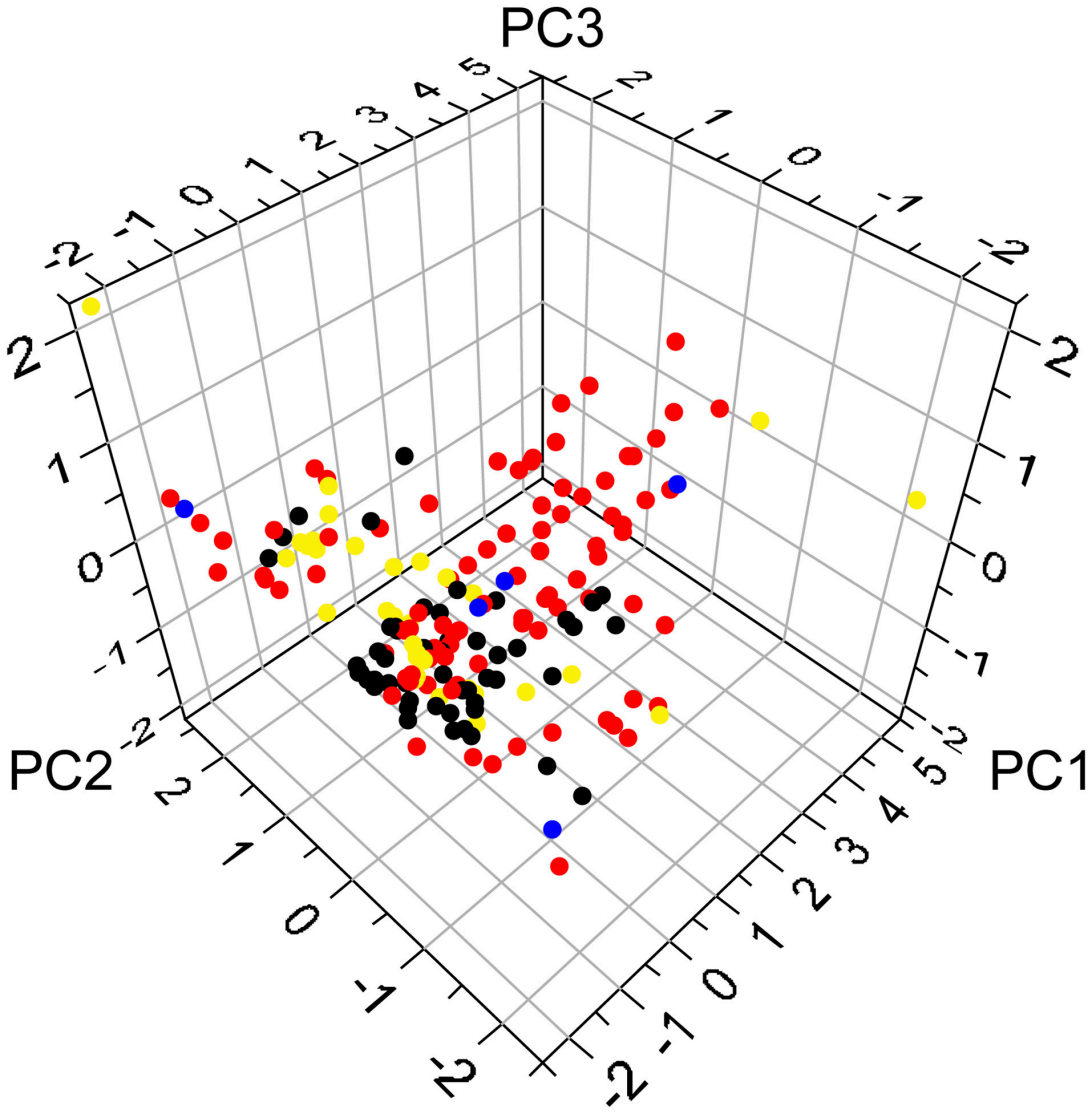
The chemical distribution according to principal component analysis. The red and black circles represent ingredients of Huangqi and Huanglian, respectively, while the blue circles delineate common ingredients of Huangqi and Huanglian. The yellow circles stand for antidiabetic drugs from DrugBank.

All of the constituent data of Huangqi and Huanglian were retrieved from TCM Systems Pharmacology Database and Analysis Platform (TcmSP™, http://ibts.hkbu.edu.hk/LSP/tcmsp.php) (Ru et al., 2014), and then manually supplemented through a wide-scale text-mining method. Meanwhile, four important pharmacology-related properties were also obtained from TcmSP™, including MW, CLogP, nHDon, and nHAcc. The principal component analysis (PCA) of the chemical distribution of Huangqi and Huanglian was built with the above four properties using the SIMCAP+ software package (version 11.0, Umetrics). The variances of PC1, PC2, and PC3 in Figure 1 account for 0.71, 0.23, and 0.04, respectively. The PCA of 34 known drug/drug-like compounds retrieved from DrugBank (http://www.drugbank.ca/) was performed in the same process as above (Supplementary Table S1).

### Active ingredients screening

The active ingredients from Huangqi and Huanglian were mainly filtered by integrating oral bioavailability (OB) and drug-likeness (DL). A robust *in silico* model OBioavail 1.1 that integrated the metabolism (P450 3A4) and transport (P-glycoprotein) information was employed to calculate the OB values of all herbal ingredients (Xu et al., 2012). Those ingredients with OB ≥ 30% were selected. Database-dependent DL evaluation approach based on Tanimoto coefficient (Ma et al., 2011) was applied and shown as *T*(*A, B*) = (*A*×*B*)/(|*A*|^2^+|*B*|^2^−*A*×*B*). In this equation, A represents the molecular descriptors of herbal compounds, and B displays the average molecular properties of all compounds in DrugBank. Those ingredients with DL ≥ 0.18 were preserved. In this study, the ingredients were adopted as the candidate compounds for further analysis when they met both of these criteria. Besides, owing to the profound pharmacological effects and high contents, those compounds with low OB or DL values were also selected for further research.

### Targets prediction

To identify the corresponding targets of the active ingredients of Huangqi and Huanglian, several approaches combined with chemometric method, information integration, and data-mining were implemented. First of all, the active ingredients were submitted to various servers viz. DRAR-CPI (Luo et al., 2011), Similarity Ensemble Approach (SEA, http://sea.bkslab.org/) (Keiser et al., 2007), STITCH (http://stitch.embl.de/) (Kuhn et al., 2012), and PharmMapper server (Wang et al., 2017). All active compounds were also sent to Herbal Ingredients' Targets database (HIT) (Ye et al., 2011), Therapeutic Targets Database (TTD, http://bidd.nus.edu.sg/group/ttd/) (Zhu et al., 2012), BindingDB database (http://www.bindingdb.org/bind/index.jsp) (Gilson et al., 2016), DrugBank and Google Scholar to mine C-T, interactions supported by literature. Then, to better dissect the role of Huangqi and Huanglian in DM treatment, all targets obtained from the previous two steps were sent to TTD, Comparative Toxicogenomics Database (CTD, http://ctdbase.org/), Online Mendelian Inheritance in Man (OMIM) database (http://www.omim.org/) and PharmGKB (http://www.pharmgkb.org) (Whirl-Carrillo et al., 2012) to mine whether these targets are related to DM. Noteworthy, only the targets of Homo sapiens were kept for further analysis.

### Gene ontology (GO) and target organ location analysis

The Molecule Annotation System 3.0 (MAS 3.0, http://bioinfo.capitalbio.com/mas3/) and DAVID (The Database for Annotation, Visualization and Integrated Discovery, http://david.abcc.ncifcrf.gov) webservers were employed to perform GO enrichment analysis for the 50 genes targeted by Huangqi and Huanglian (Huang da et al., 2009). The target organ distribution was determined based on the microarray analyses data of different tissue types lodged in the BioGPS bank (http://biogps.org) (Wu et al., 2009).

### Network construction

Three visualized networks were constructed: (1) Compound-Target network (C-T network). Active ingredients of Huangqi and Huanglian and their corresponding targets were employed to generate the C-T network; (2) Target-Pathway network (T-P network). The pathway information of targets were extracted from KEGG (Kyoto Encyclopedia of Genes and Genomes, http://www.kegg.jp), and then a bipartite T-P network composed of targets and their corresponding putative pathways was built; (3) T-O network. The potential targets and their tissue types were applied to T-O network. All visualized networks were constructed by Cytoscape 3.5.1 (http://www.cytoscape.org/), an open software package project for visualizing, integrating, modeling and analyzing the interaction networks (Smoot et al., 2011).

### Contribution indexes calculation

In order to estimate the contribution of each active ingredient to the antidiabetic effects of Huangqi and Huanglian, a contribution index (CI) based on network based efficacy (NE) weighted by literature was calculated by Equation (1) and equation (2) (Yue et al., 2017):

(1) $$\begin{array}{rcl} NE\left(j\right) & = & \overset{n}{\underset{i=1}{\sum }}d_{i} \end{array}$$

(2) $$\begin{array}{rcl} CI\left(j\right) & = & \frac{c_{j}\times NE\left(j\right)}{\overset{m}{\underset{i=1}{\sum }}c_{i}\times NE\left(i\right)}\times 100\% \end{array}$$

Where *n* is the number of targets associated with ingredient *j* in the C-T network; *d*_*i*_ is the degree of target *i* associated with ingredient *j* in the T-P network; *c*_*i*_ is the number of DM-related literature of ingredient *i*; *m* is the number of ingredients. For DM-related literature-mining approach, the following keywords were used for DM terms: diabetes, hyperglycemia, and insulin resistance and the common names of active ingredients were also used as search keywords. The numbers of papers having keywords in the title or abstract published in 1990–2017 were obtained from the PubMed database. If the sum of CIs for the top *N* ingredients was more than 85%, these relevant *N* ingredients were considered to contribute the most to the antidiabetic effects.

## Results

### Chemical distribution of huangqi and huanglian

The ingredients in Huangqi and Huanglian were retrieved from TcmSP™ and published literatures. Since those glycosides in Huangqi and Huanglian might be deglycosylated in the intestinal tract associated with gut microbiota, 13 aglycones were also incorporated into the compound library labeled by _qt. Thus, a total of 171 ingredients were retrieved for Huangqi (112) and Huanglian (64), including five common ingredients. The detailed information about these molecules was provided in Supplementary Table S2.

The PCA was further conducted to give visual illustration in chemical distribution. The constituents of Huangqi and Huanglian were diverse and both of them possessed a broad diversity in chemical space (Figure 1), but the majority of them satisfied the Lipinski's rule of five. Moreover, the large overlap between the ingredients in Huangqi and Huanglian and 34 known drug/drug-like compounds for DM demonstrated that many compounds contained in these two herbs had drug potential on DM. Noticeably, since PC2 was highly associated with the value of nHDon, those ingredients of Huangqi (red circle) including astragalosides with PC2 in the range 3~5 possessed larger nHDon values than the antidiabetic drugs from DrugBank (yellow circle), whereas the ingredients of Huanglian (black circle) overlap better with the antidiabetic drugs in Figure 1. Thus, it is meaningful to figure out these active ingredients in Huangqi and Huanglian for DM treatment.

### Active ingredients in huangqi and huanglian

Although a single herb or TCM formula usually contains large numbers of compounds, virtual screening approaches are always of great help to distinguish those active ingredients. In the present work, two ADME (absorption, distribution, metabolism, and excretion)-related models, including OB and DL were employed to screen most of the active ingredients from Huangqi and Huanglian. A few of active compounds that do not meet either of these two criteria were also selected in the cases of high bioactivities and huge amounts. Consequently, a total of 43 active compounds were selected from the 171 compounds of these two herbs (Table 1).

**Table 1:** Active ingredients and ADME parameters of Huangqi and Huanglian.

**No**.	**Name**	**Structure**	**OB (%)**	**DL**	**Herb**
M1^a^	Berberine	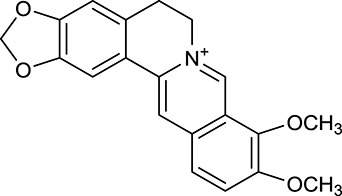	068	078	*C. chinensis*
M2^a^	Columbamine	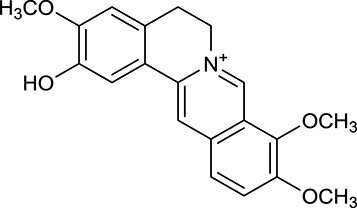	2694	059	*C. chinensis*
M3	Berberrubine	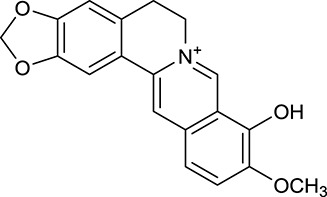	3574	073	*C. chinensis*
M4	8-Oxocoptisine	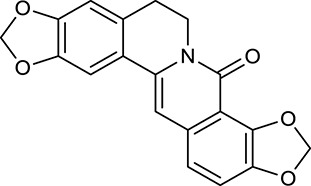	4683	089	*C. chinensis*
M10^a^	Magnoflorine	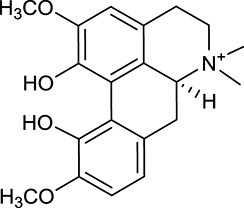	2260	055	*C. chinensis*
M12	Epiberberine	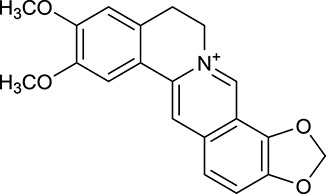	4309	078	*C. chinensis*
M13^a^	Groenlandicine	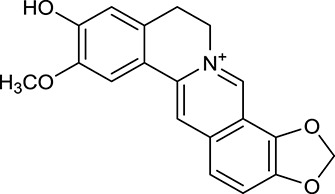	2842	072	*C. chinensis*
M16^a^	Phellodendrine	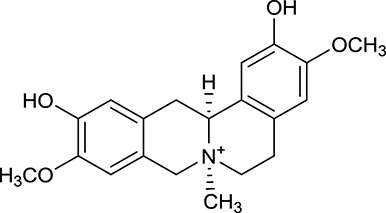	250	058	*C. chinensis*
M18	*(R)*-Canadine	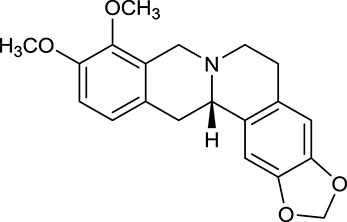	5537	077	*C. chinensis*
M19	Berlambine	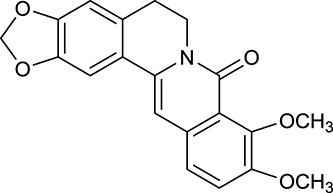	3668	082	*C. chinensis*
M20^a^	Jatrorrhizine	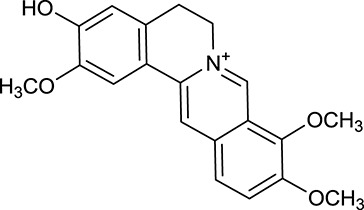	1965	059	*C. chinensis*
M21	Palmatine	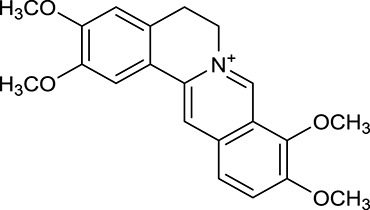	6460	065	*C. chinensis*
M23^a^	Coptisine	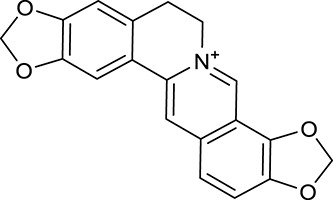	721	086	*C. chinensis*
M26	Worenine	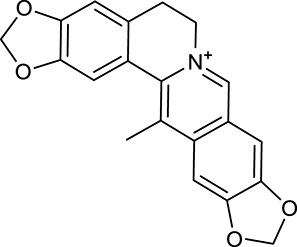	45.83	0.87	*C. chinensis*
M27	Obacunone	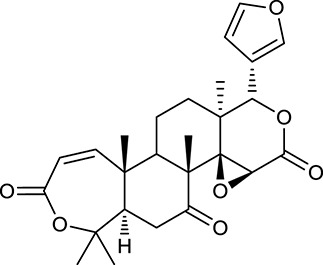	43.29	0.77	*C. chinensis*
M32^a^	Ferulic acid	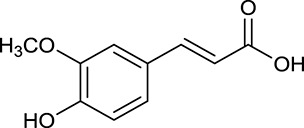	39.56	0.06	*C. chinensis*
M33^a^	Vanillic acid	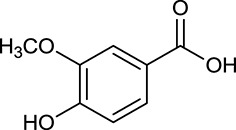	35.47	0.04	*C. chinensis*
M60	β-Sitosterol	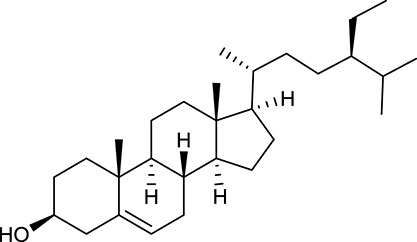	36.23	0.78	*C. chinensis*/*A. membranaceus*
M61	Hederagenin	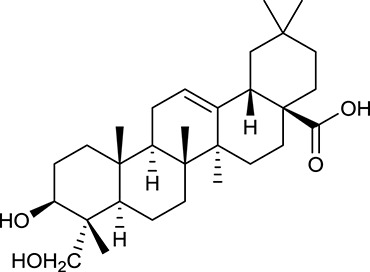	36.91	0.75	*A. membranaceus*
M62^a^	Lupeol	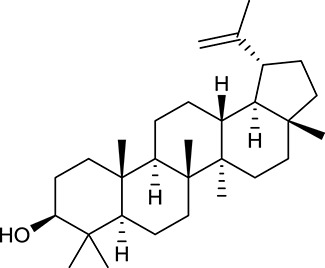	12.12	0.78	*A. membranaceus*
M85^a^	Soyasaponin I	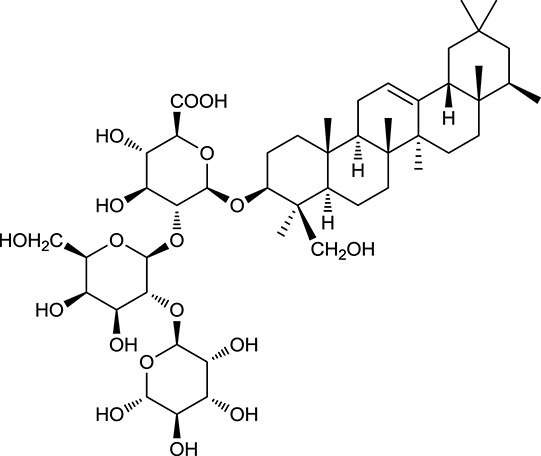	2.06	0.15	*A. membranaceus*
M92^a^	Astragaloside I	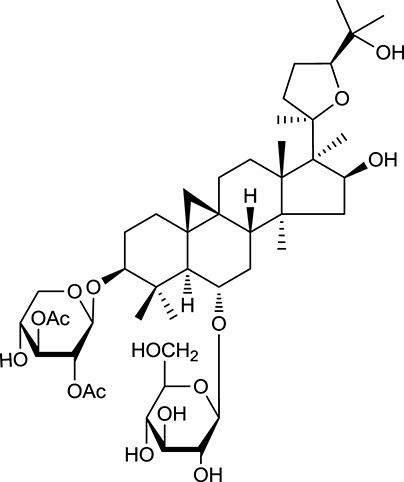	46.79	0.11	*A. membranaceus*
M94^a^	Astragaloside II	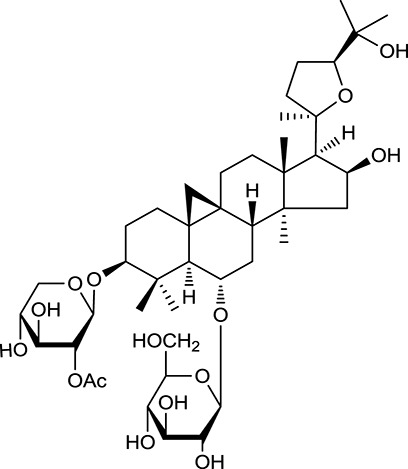	0.79	0.13	*A. membranaceus*
M96^a^	Astragaloside III	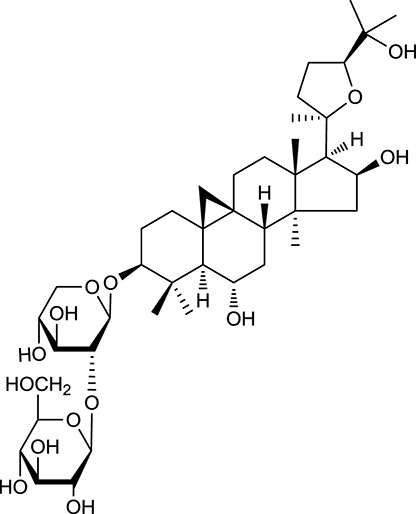	31.83	0.10	*A. membranaceus*
M98^a^	Astragaloside IV	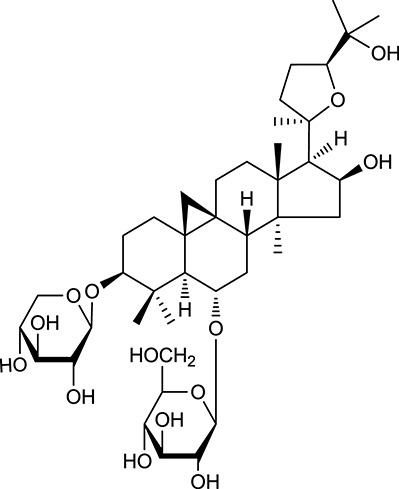	2.20	0.15	*A. membranaceus*
M104^a^	Isoastragaloside I	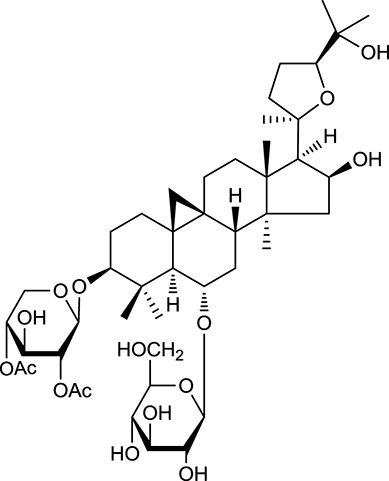	37.80	0.14	*A. membranaceus*
M109^a^	Cycloastragenol	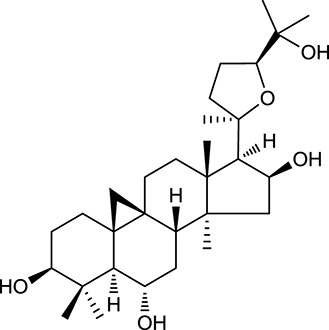	25.70	0.10	*A. membranaceus*
M115	Kumatakenin	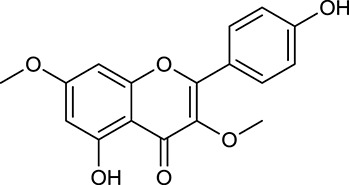	50.83	0.29	*A. membranaceus*
M118	Isorhamnetin	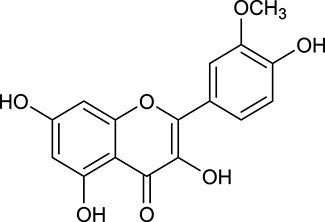	4960	031	*A. membranaceus*
M119	3,9-di-*O*-Methylnissolin	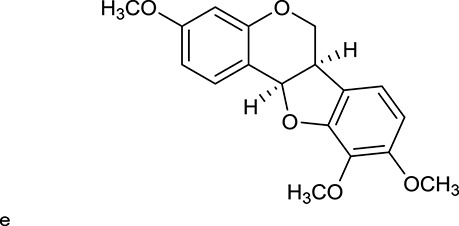	5374	048	*A. membranaceus*
M120	Calycosin	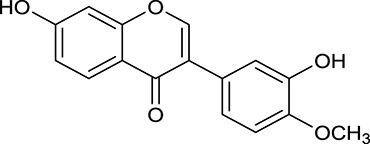	47.75	0.24	*A. membranaceus*
M121^a^	Calycosin 7-*O*-β-D-glucopyranoside	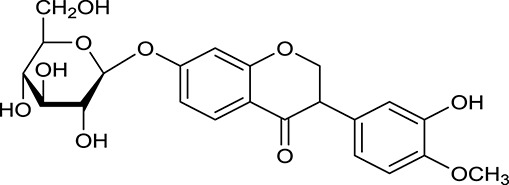	1005	081	*A. membranaceus*
M122	7-*O*-Methylisomucronulatol	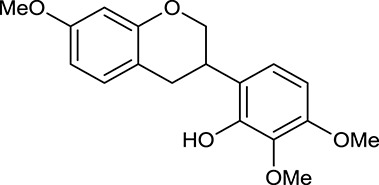	7469	030	*A. membranaceus*
M123	(6α*R*, 11α*R*) 3-Hydroxy-9,10-dimethoxypterocarpan-3-*O-β-*D-glucoside	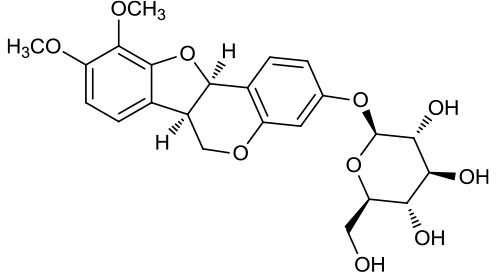	36.74	0.92	*A. membranaceus*
M124	(6α*R*, 11α*R*) 3-Hydroxy-9,10-dimethoxypterocarpan	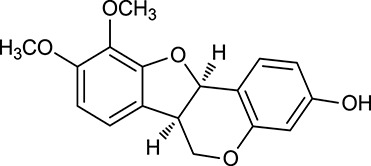	64.26	0.42	*A. membranaceus*
M125	Formononetin	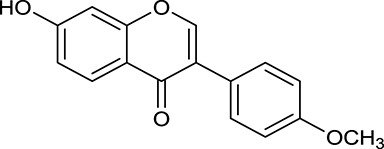	69.67	0.21	*A. membranaceus*
M126^a^	Formononetin-7-*O*-β-D-glucoside	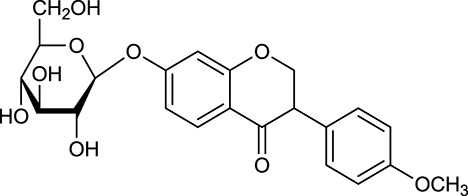	1152	078	*A. membranaceus*
M127^a^	Rhamnocitrin-3-*O*-glucoside	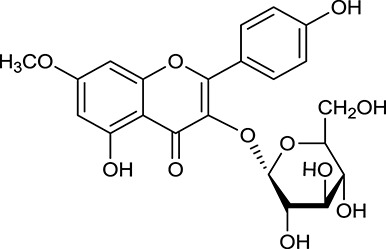	2.87	0.76	*A. membranaceus*
M132	Isomucronulatol	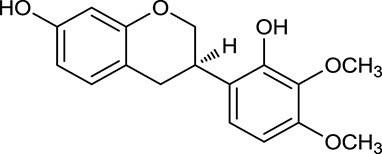	67.67	0.26	*A. membranaceus*
M141^a^	Rutin	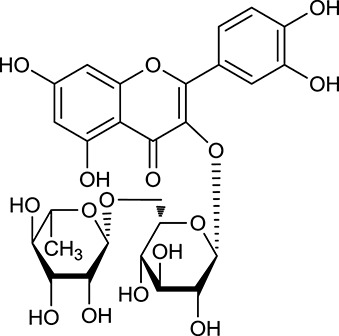	11.70	0.68	*C. chinensis/A. membranaceus*
M148	Quercetin	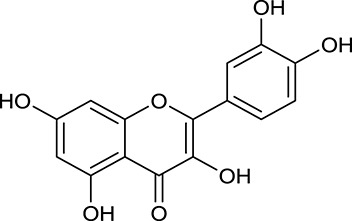	4643	028	*C. chinensis*/*A. membranaceus*
M154	Kaempferol	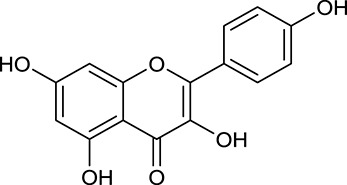	67.43	0.24	*C. chinensis/A. membranaceus*
M171^a^	Astragalus polysaccharides	N/A	N/A	N/A	*A. membranaceus*

*OB, oral bioavailability; DL, druglikeness; A. membranaceus, Astragalus membranaceus (Huangqi); C. chinensis, Coptis chinensis (Huanglian)*.

a *Compounds with OB < 30% and/or DL < 0.18, yet validated pharmaceutically*.

### Active ingredients from huangqi

In Huangqi, only 26 ingredients passed through the strict filtering criteria, and most of them exhibited potent pharmacological activities. For examples, calycosin (M120, OB = 47.75%, and DL = 0.24) displayed therapeutic effects on diabetic complication (Xu et al., 2011); formononetin (M125, OB = 69.67%, and DL = 0.21) showed significant antihyperglycemic activity (Qiu et al., 2017). Other characteristic isoflavonoids in Huangqi accounted for large contents and were also preserved. Specifically, calycosin-7-*O*-β-D-glucoside (M121, OB = 10.05%, and DL = 0.81), formononetin-7-*O*-β-D-glucoside (M126, OB = 11.52%, and DL = 0.78), (6α*R*, 11α*R*) 3-hydroxy-9,10-dimethoxypterocarpan-3-*O-*β*-*D-glucoside (M123, OB = 36.74%, and DL = 0.92), together with calycosin and formononetin in Huangqi were measured up to 0.44–1.76 mg/g (Wu et al., 2005). Surprisingly, astragalosides, the main active and characteristic compounds in Huangqi, exhibited low OB values (Gu et al., 2004; Ma et al., 2017). Among them, astragaloside IV (AIV, M98, OB = 2.20%, and DL = 0.15) has been determined as the quality marker of Huangqi in *Chinese Pharmacopoeia* (The State Pharmacopoeia Commission of China, 2015) and exhibited significant hypoglycemic effect (Lv et al., 2010). Astragaloside II (M94, OB = 0.79%, and DL = 0.13) and isoastragaloside I (M104, OB = 37.80%, and DL = 0.14) could alleviate insulin resistance and glucose intolerance by enhancing the expression of an insulin-sensitizing adiponectin (Xu et al., 2009). Besides, the contents of astragaloside I (M92, OB = 46.79%, and DL = 0.11), astragaloside II, astragaloside III (M96, OB = 31.83%, and DL = 0.10) and AIV in Huangqi were 0.78, 0.35, 0.20, and 0.26 mg/g, respectively (Zu et al., 2009). Importantly, astragalus injection (the major components are astragalosides) has been used in China to treat DM with good clinical effects (Nie et al., 2014). Thus, astragalosides were also selected for targeting. Astragalus polysaccharides (M171) as a polysaccharide mixture from Huangqi (accounted for 90.00–288.75 mg/g) was also preserved because it could ameliorate insulin resistance and restore glucose homeostasis, in part, via gut microbiota (Zou et al., 2009; Zhang et al., 2013; Liu et al., 2014).

#### Active ingredients from huanglian

By ADME screening, 21 out of 64 ingredients with excellent pharmacological effects were extracted from Huanglian, and half of them possessed satisfactory pharmacokinetic profiles. It should be pointed out that most of the representative isoquinoline alkaloids in Huanglian showed extremely low OB values (Li et al., 2006; Chen et al., 2011; Bao et al., 2015), but they exhibited potent antidiabetic, anti-inflammatory, and antioxidant activities (Patel and Mishra, 2012; Zhang et al., 2012). For instances, berberine (BBR, M1, OB = 0.68%, and DL = 0.78) had lipid- and glucose-lowing effects in treatment of DM and obesity, which may be associated with gut microbiota (Zhang et al., 2012); magnoflorine (M10, OB = 22.60%, and DL = 0.55) could exert antioxidant and antiglycemic effects *in vivo* (Patel and Mishra, 2012). Besides, BBR and coptisine (M23, OB = 7.21%, and DL = 0.86) together with epiberberine (M12, OB = 43.09%, and DL = 0.78) and palmatine (M21, OB = 64.60%, and DL = 0.65) have been chosen as the marker components for quality control of Huanglian in *Chinese Pharmacopoeia* (The State Pharmacopoeia Commission of China, 2015). Meanwhile, the isoquinoline alkaloids accounted for large amounts in Huanglian. For example, the contents of BBR, coptisine, jatrorrhizine (M20, OB = 19.65%, and DL = 0.59), palmatine, and epiberberine ranged from 58.47 to 71.35, 19.38 to 22.56, 4.96 to 5.33, 15.68 to 20.57, and 11.05 to 12.44 mg/g, respectively, and the total content of these five alkaloids was 111.33–129.42 mg/g (Ding et al., 2012). In view of the facts mentioned above, isoquinoline alkaloids were deemed as the active ingredients for further analysis. Strikingly, ferulic acid (M32, OB = 39.56%, and DL = 0.06) as a minor constituent in Huanglian exhibited synergistic effect on antihyperglycemic activity in combination with BBR (Chen et al., 2012). Vanillic acid (M33, OB = 35.47%, and DL = 0.04) has the potential to prevent the progression of DM via ameliorating insulin resistance (Chang et al., 2015). It was reasonable to believe that the above compounds could be listed as potential active ingredients for Huanglian (Table 1).

Noteworthy, besides choline, other four shared compounds were selected and have beneficial effects on DM. Specifically, β-sitosterol (M60, OB = 36.23%, and DL = 0.78) exhibited potent antidiabetic and antioxidant activities (Gupta et al., 2011); rutin (M141, OB = 11.70%, and DL = 0.68) had hypoglycemic effect and may confer protective effects against diabetic nephropathy (Han et al., 2017); quercetin (M148, OB = 46.43%, and DL = 0.28) presented anti-obesity and antidiabetic activities (Aguirre et al., 2011); additionally, kaempferol (M154, OB = 67.43%, and DL = 0.24) could exert antidiabetic benefits through protecting beta-cells against glucotoxicity (Zhang and Liu, 2011).

### Target proteins of huangqi and huanglian

Searching for the targets of candidate drugs solely by the experimental approaches is overspending, labor-intensive, and time-consuming. In the present work, an integrated *in silico* approach was introduced to identify the target proteins for the active ingredients of Huangqi and Huanglian. Predictive models were used including DRAR-CPI, SEA, STITCH and PharmMapper server, and databases were mined including HIT, TTD, BindingDB database, DrugBank and Google Scholar. Finally, 50 DM-related targets were determined, interacting with the selected 43 active ingredients of this herb combination (Table 2). Of note, we have implemented molecular docking and surface plasmon resonance (SPR) assay to explore the reliability of the interactions between the active ingredients and their putative targets. As shown in Supplementary Table S3, Supplementary Figures S3, S4, it could be concluded that the predicted targets were convincible to explore the network interactions of Huangqi and Huanglian.

**Table 2:** Target information of Huangqi and Huanglian.

**ID**	**Target**	**UniProt ID**	**Gene name**
T-01	Estrogen receptor	P03372	ESR1
T-02	Estrogen receptor beta	Q92731	ESR2
T-03	Peroxisome proliferator-activated receptor alpha	Q07869	PPARA
T-04	Peroxisome proliferator activated receptor gamma	P37231	PPARG
T-05	Superoxide dismutase [Cu-Zn]	P00441	SOD1
T-06	Hepatocyte nuclear factor 4-alpha	P41235	HNF4A
T-07	Prostaglandin G/H synthase 2	P35354	PTGS2
T-08	Calmodulin-1	P0DP23	CALM1
T-09	5-Hydroxytryptamine 2C receptor	P28335	HTR2C
T-10	Vascular endothelial growth factor A	P15692	VEGFA
T-11	Nitric oxide synthase, inducible	P35228	NOS2
T-12	Nitric oxide synthase, endothelial	P29474	NOS3
T-13	Glucocorticoid receptor	P04150	NR3C1
T-14	Interleukin-1 beta	P01584	IL1B
T-15	Heme oxygenase 1	P09601	HMOX1
T-16	Phosphatidylinositol-4,5-bisphosphate 3-kinase catalytic subunit, gamma isoform	P48736	PIK3CG
T-17	Tumor necrosis factor	P01375	TNF
T-18	Glutathione S-transferase Mu 1	P09488	GSTM1
T-19	Acetylcholinesterase	P22303	AChE
T-20	Caspase-3	P42574	CASP3
T-21	Caspase-9	P55211	CASP9
T-22	mRNA of protein-tyrosine phosphatase, non-receptor type 1	P18031	PTP1B
T-23	Glucagon-like peptide 1 receptor	P43220	GLP1R
T-24	Aldose reductase	P15121	AKR1B1
T-25	Solute carrier family 2, facilitated glucose transporter member 2	P11166	GLUT2
T-26	Solute carrier family 2, facilitated glucose transporter member 4	P14672	GLUT4
T-27	Interleukin-2	P60568	IL2
T-28	Interleukin-6	P05231	IL6
T-29	Hepatocyte nuclear factor 1-alpha	P20823	HNF1A
T-30	Glucokinase	P35557	GCK
T-31	Insulin-degrading enzyme	P14735	IDE
T-32	Insulin-like growth factor 1 receptor	P08069	IGF1R
T-33	Insulin receptor	P06213	INSR
T-34	Lysosomal alpha-glucosidase	P10253	GAA
T-35	Phosphatidylinositol 3-kinase regulatory subunit alpha	P27986	PIK3R1
T-36	Dipeptidyl peptidase IV	P27487	DPP4
T-37	C-C motif chemokine 2	P13500	CCL2
T-38	Glycogen synthase kinase-3 beta	P49841	GSK3B
T-39	Glutathione S-transferase Mu 2	P28161	GSTM2
T-40	Mitogen-activated protein kinase 1	P28482	MAPK1
T-41	Mitogen-activated protein kinase 14	Q16539	MAPK14
T-42	Beta-2 adrenergic receptor	P07550	ADRB2
T-43	NAD(P)H dehydrogenase [quinone] 1	P15559	NQO1
T-44	78 kDa glucose-regulated protein	P11021	HSPA5
T-45	Glycogen phosphorylase	P06737	PYGL
T-46	Farnesoid X receptor	B6ZGS9	FXR
T-47	5′-AMP-activated protein kinase catalytic subunit alpha-2	P54646	AMPK
T-48	Pancreatic α-amylase	P04746	AMY2A
T-49	Cytochrome P450 3A4	P08684	CYP3A4
T-50	Cyclin-dependent kinase 2	P24941	CDK2

#### Target proteins of huangqi

For Huangqi, by target fishing, 26 active ingredients were validated to bind with 42 DM-related target proteins. For example, AIV may have the potential to act on 21 targets including NR3C1, VEGFA, NOS2, CASP3, PYGL, PPARG, and AKR1B1. Actually, AIV has been identified as an inhibitor of NR3C1, which might contribute to its therapeutic application in DM (Liu et al., 2016). Beyond that, it also has a strong antagonistic effect on PYGL, suggestive of its contribution to the control of blood glucose homeostasis (Lv et al., 2010). Analogously, formononetin and calycosin exhibited strong activation on PPARA and PPARG to correct dyslipidemia and to restore glycemic balance (Shen et al., 2006). Formononetin could also alleviate the retinal neovascularization of diabetic retinopathy by inhibiting VEGFA (Wu et al., 2016). Cycloastragenol, as the aglycone of astragalosides could improve hepatic steatosis through activating FXR, thereby alleviating DM-related hyperglycemia and hyperlipidemia (Gu et al., 2017). Hederagenin and lupeol interacted with GSTM1 to decrease the risk of diabetic retinopathy and nephropathy (Datta et al., 2010; Sun et al., 2015). Notably, although it is hardly to predict the targets of astragalus polysaccharides *in silico*, some DM-related targets, such as GAA, PTP1B, and AMPK, have been reported to be associated with its antidiabetic effect (Zou et al., 2009; Zhao et al., 2012; Zhu et al., 2014).

#### Target proteins of huanglian

Forty-three targets were identified for 21 active ingredients of Huanglian with 318 interactions, such as PIK3CG, PTP1B, AMPK, PIK3R1, IL1B, HNF4A, and MAPK1. For instance, BBR, epiberberine, magnoflorine, and coptisine inhibited PTP1B to increase insulin and leptin activities, thereby possibly exerting antidiabetic activity (Choi et al., 2015). Vanillic acid may interact with four potential targets including GLUT2, IDE, GLP1R, and GAA to display antidiabetic activity (Chang et al., 2015). It was worthy to mention that some major targets, such as AKR1B1, ADRB2, HMOX1, and NOS3, were also closely concerned with the various symptoms of diabetic complications. BBR, epiberberine, coptisine, and groenlandicine could inhibit AKR1B1 to alleviate the diabetic complications (Jung et al., 2008; Liu et al., 2008). BBR, palmatine and (*R*)-canadine may interact with ADRB2 to prevent the progression of obesity and hypertriglyceridaemia (Ishiyama-Shigemoto et al., 1999).

### GO enrichment analysis for targets

**Figure 2:**
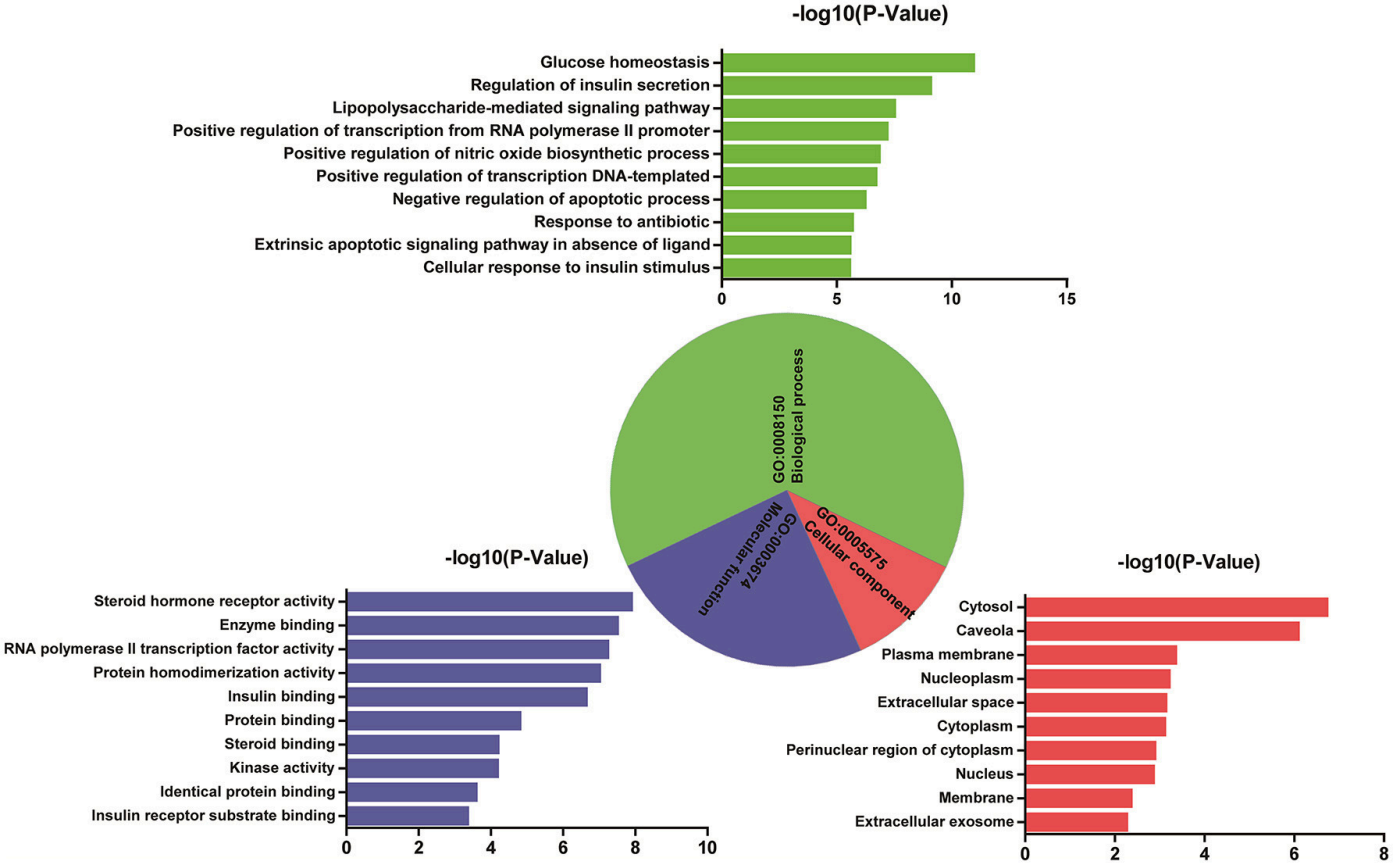
GO enrichment analysis of the targets of Huangqi and Huanglian. Biological process (green), molecular function (blue), and cellular component (red) accounted for 64.82, 24.10, and 11.08%, respectively.

Analysis of interaction network regulation of 50 targets was performed using MAS 3.0. As shown in Figure 2, biological process (BP, GO:0008150), molecular function (MF, GO:0003674), and cellular component (CC, GO:0005575) accounted for 64.82, 24.10, and 11.08%, respectively. Further, BP, MF, and CC enrichment analysis were performed by DAVID bioinformatics resources. The top 10 significantly enriched terms in BP, MF, and CC categories (*P* < 0.05, *P*-values were corrected using the Benjamini-Hochberg procedure) were listed in Figure 2, indicating that Huangqi and Huanglian may regulate glucose homeostasis, insulin secretion, and nitric oxide biosynthetic process via enzyme binding, insulin binding, and kinase binding in the cytosol, caveola, and plasma membrane so as to exert antidiabetic potential. It is interesting to note that a large number of targets were associated with a variety of BP terms such as regulation of insulin secretion, glucose homeostasis, cellular response to insulin stimulus, lipopolysaccharide-mediated signaling pathway, and positive regulation of nitric oxide biosynthetic process, which are closely related to the pathogenesis of DM. KEGG pathway enrichment analysis was also performed by DAVID bioinformatics resources and displayed in Supplementary Figure S5. Furthermore, network and functional association of 50 targets of Huangqi and Huanglian was mapped in Supplementary Figure S6 using GeneMANIA (http://www.genemania.org/).

### Network analysis to decipher the synergistic mechanisms of huangqi and huanglian

#### Compound-target network analysis

**Figure 3:**
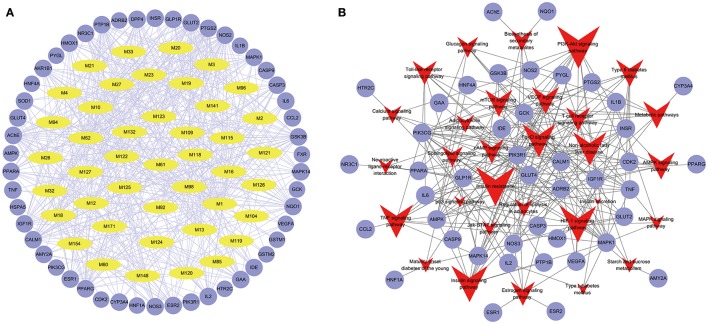
Compound-Target **(A)** and Target-Pathway **(B)** networks of Huangqi and Huanglian. The yellow and light blue nodes are active ingredients and their potential targets of Huangqi and Huanglian, while the red nodes represent the pathways.

To facilitate the visualization and interpretation of the complex relationships between all active ingredients of Huangqi and Huanglian and their targets, a bipartite graph of C-T network was constructed (Figure 3A). All active ingredients in these two herbs were potential multiple-kinase inhibitors or activators. Amongst them, those ones with high interconnection degrees were responsible for the high interconnectedness of the C-T network, especially BBR (degree = 33), lupeol (degree = 22), quercetin (degree = 22), AIV (degree = 21), epiberberine (degree = 21), calycosin (degree = 21), and (*R*)-canadine (degree = 21). Importantly, as shown in the C-T network (Figure 3A), the efficacy of this herb combination not only concentrated on modulating the crucial targets involving in the glucose and insulin homeostasis (IGF1R, GAA, IDE, HNF1A, GCK, and DPP4), but also, more essentially, focused on the regulation of the other proteins mediating diabetic complications including inflammation, retinopathy, neuropathy, nephropathy, and abdominal pain (NOS2, AKR1B1, VEGFA, PTGS2, ESR2, and AChE) to relieve the pathological changes and prolong the efficient curing process. Additionally, as expected, the majority of the targets (32) such as GLUT2, NOS2, PTP1B, and IGF1R were synergistically regulated by different components of Huanglian and Huangqi.

#### Target-pathway network analysis

**Figure 4:**
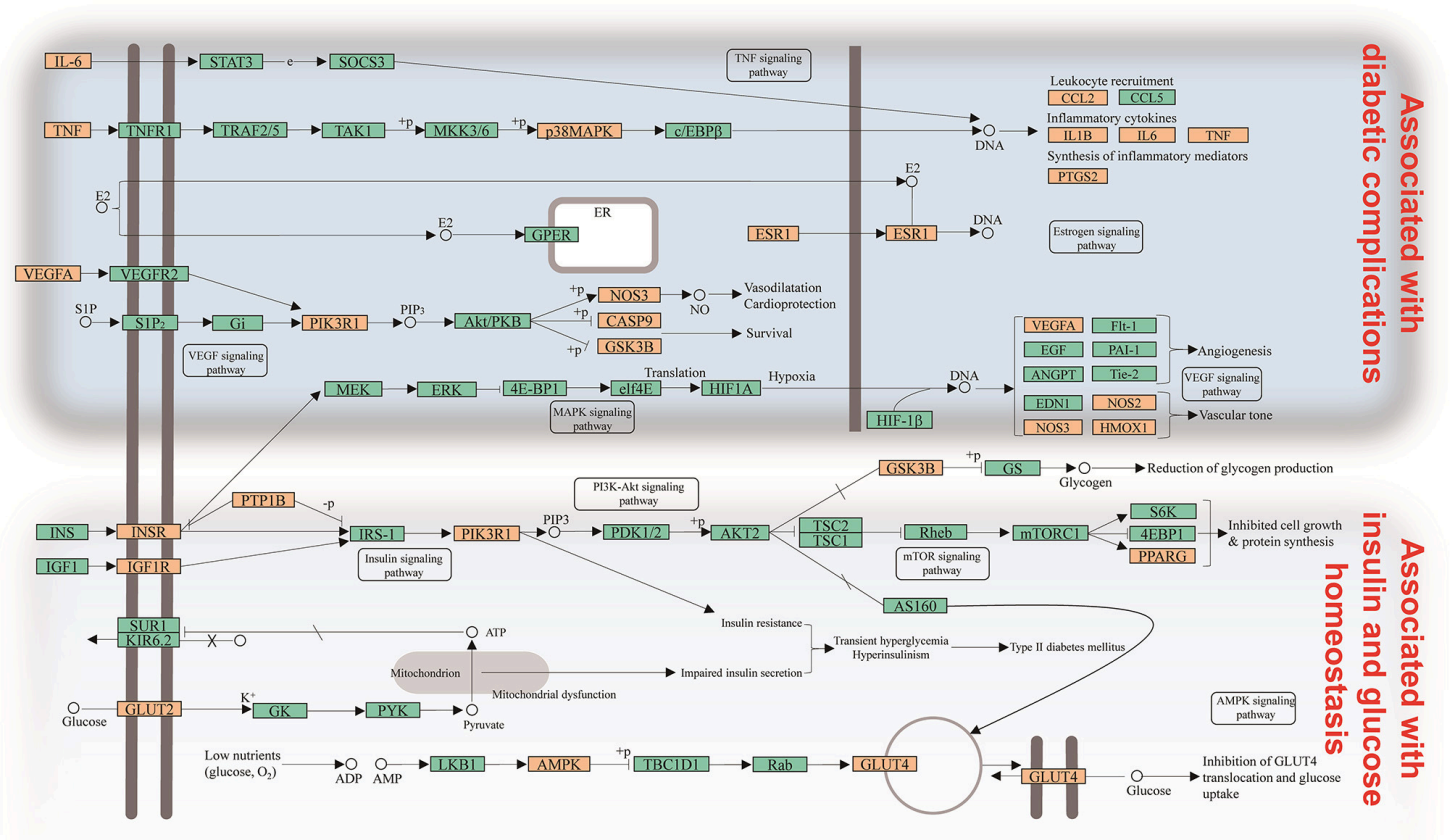
Distribution of partial targets of Huangqi and Huanglian on the compressed pathway. The orange nodes are potential targets of Huangqi and Huanglian, while the light blue nodes are relevant targets in the pathway.

Signaling pathways, as an important component of the system pharmacology, link receptor-ligand interactions to pharmacodynamics outputs. All of the targets interacting with the active ingredients were mapped onto the 30 KEGG pathways and the T-P network was generated (Figure 3B). The PI3K-Akt signaling pathway exhibited the highest number of target connections (degree = 13), followed by insulin resistance with 12 targets, insulin signaling pathway and HIF-1 signaling pathway with 11 ones, respectively. These high-degree pathways have well-established roles in the insulin secretion and glucose homeostasis (Taniguchi et al., 2006). Besides, the VEGF signaling pathway played an important role in the diabetic retinopathy involved in multiple targets including PIK3CG, PTGS2, and VEGFA (Antonetti et al., 2012). The activation of mTOR signaling pathway was an underlying cause of renal hypertrophy at the early stage of DM (Sakaguchi et al., 2006). Interestingly, we also found that the combination of Huangqi and Huanglian may exert its therapeutic effects on DM by regulating the pathways related to the adipogenesis and/or lipolysis in adipocytes, liver, and vascular tissues. As shown in the compressed pathway (Figure 4), Huangqi and Huanglian synergistically acted on the multiple targets in these high-degree pathways.

**Figure 5:**
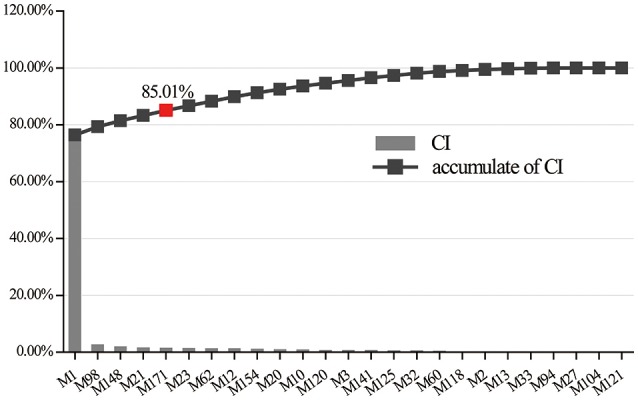
The CI and accumulative CI of active ingredients in Huangqi and Huanglian. The sum of CIs for the top five ingredients including M1 (BBR), M98 (AIV), M148 (quercetin), M21 (palmatine), and M171 (astragalus polysaccharides) was more than 85%.

Integrating with the above two networks, a CI of every active ingredient was proposed based on NE weighted by literature (Figure 5, Supplementary Table S4). Five compounds emerged from the active ingredients, including BBR, AIV, quercetin, palmatine, and astragalus polysaccharides. They displayed the most contribution to the antidiabetic effects of Huangqi and Huanglian with a sum of CIs of 85.01%. Therefore, the above discussion may fully clarify why Huangqi and Huanglian could produce synergistic and complementary effects.

#### Target-organ network analysis

**Figure 6:**
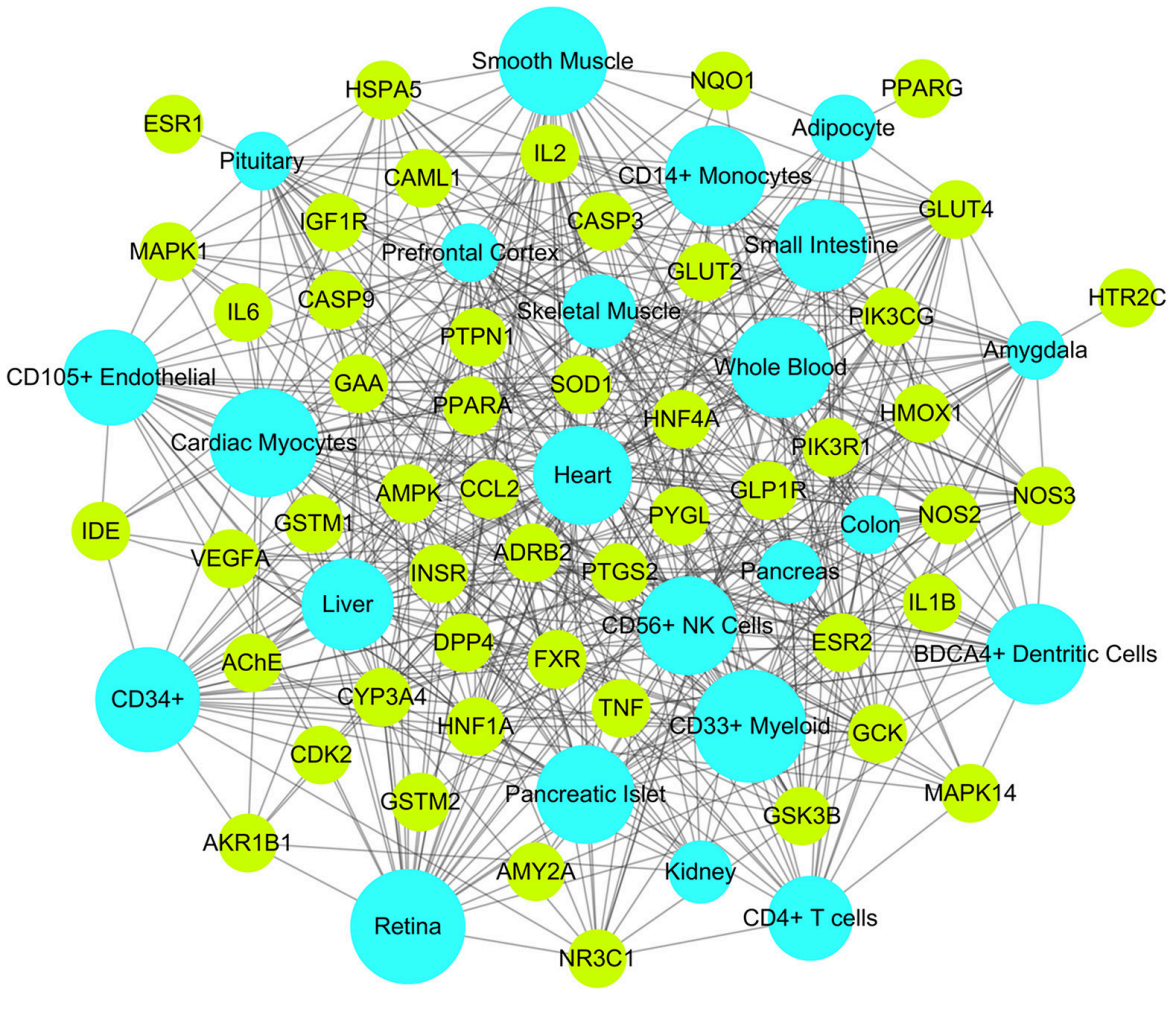
Target-Organ network of Huangqi and Huanglian where kelly nodes represent the targets and light blue nodes represent the organs.

It may facilitate the development of enhanced detection and treatment modalities for DM by understanding how the multi-organs respond to indications on a system level. We compared the expression patterns of 50 targets across different tissues at different levels according to the BioGPS database. The tissue distribution network of the 50 targets were mapped and shown in Figure 6. Most targets acted on two or more tissues, suggesting that these tissues are closely correlated. Specifically, 30 targets contained high mRNA expression in retina, accounting for 60% of all the targets. There were 29 targets (accounting for 58% of all the targets) located in the CD33 + myeloid, suggesting they were potential effective targets for the treatment of autoimmune DM. In addition, 20 targets were overexpressed in small intestine and 15 targets in colon. It is evident that patients with DM have a high incidence of gastrointestinal dysfunction (Abrahamsson, 1995) and gut microbiota disturbance (Kootte et al., 2012). Similarly, 27 targets acted in cardiac myocytes and 22 targets in heart, consistent with the fact that DM increases coronary heart disease (CHD) morbidity and mortality and is considered a CHD risk equivalent (Newman et al., 2017). Noticeably, the targets in whole blood were linked with tissues in almost all the forms, indicating that whole blood acted as the bridge and glycemic excursion played a vital role in the pathological processes of these tissues.

## Discussion

According to the TCM practice for thousands of years, DM can be classified as Xiao Ke (wasting thirst syndrome), which is usually associated with the deficiency of both *Qi* (vital energy) and *Yin* (body fluids) resulting in the *Heat* of tissues and blood or urine stasis. The “Gan” (sweetish taste) and “Ku” (bitter taste) are recognized as the popular therapeutic flavors to Xiao Ke syndrome (Xia et al., 2016). Among the commonly used herbs beneficial for DM, Huangqi (“Gan” flavor) and Huanglian (“Ku” flavor) are given high priority for selection and their combination has been frequently used in TCM prescriptions (Zhang et al., 2010; Xie et al., 2011). In our work, an integrated system pharmacology approach was successfully applied to illuminate the molecular synergy of Huangqi and Huanglian on DM. Forty-three active ingredients and 50 corresponding DM-related targets were selected and predicted, which were mainly involved in 30 KEGG signaling pathways associated with DM treatment and prophylaxis. By systematic analysis of the C-T network, the astragalosides and isoflavonoids of Huangqi may mainly stimulate insulin secretion, improve insulin resistance and promote glucose utilization, but the isoquinoline alkaloids of Huanglian could regulate inflammatory cytokines, promote the utilization of glucose, and improve endocrine and metabolism so as to achieve the synergistic and complementary curative effects of Huangqi and Huanglian.

DM is inevitably accompanied with the development of serious complications, including retinopathy, neuropathy, and nephropathy. Therefore, in clinical practice, hypoglycemic drugs are always prescribed with other drugs for complications, such as antibiotics and antiulcer agents, which may increase the risk of adverse drug events (Held et al., 2017). In our study, this herb combination could not only regulate the insulin secretion and glucose homeostasis but also inflammation and immunity. And the targets were mainly located in retina, pancreatic islet, smooth muscle, immunity-related organ tissues, and whole blood, which were highly associated with Xiao Ke, especially San Xiao syndromes. These results are expected to take full clinical advantage of Huangqi and Huanglian for diabetic complications.

Disruptions in gut microbiota composition and function are increasingly implicated in the pathogenesis of obesity, insulin resistance, and DM (Tremaroli and Bäckhed, 2012). On the other hand, gut microbiota plays a crucial role in TCM therapy by complicated interplay with herb-derived compounds, such as alkaloids, saponins, and polysaccharides (Xu et al., 2017). Therefore, the availability of these active constituents of Huangqi and Huanglian by gut microbiota especially under the DM state may be a critical step toward the emergence of their bioactivities *in vivo*.

Besides, through SPR assay, we demonstrated that calycosin and coptisine could bind with GAA, which were consistent with the previous reports (Zhou et al., 2012; Zhao et al., 2015). Astragaloside I and AIV were found to exhibit significant interaction with TNF and PTP1B, respectively, suggestive of their potential for diabetic complications. However, instead of AIV, there are very few reports that focus on the pharmacological actions of other astragalosides. Jatrorrhizine and palmatine showed a strong interaction with GLP1R; calycosin and calycosin 7-*O*-β-D-glucopyranoside were observed to bind with PPARG and INSR, respectively. Therefore, more experiments are anticipated to support our intriguing findings.

## Author contributions

CW and DY conceived of and proposed the idea. SY and JL designed the study. SY, JL, and FZ performed the experiments. SY, JL, WF, JC, and DY participated in data analysis. CW, DY, CP, and HG contributed to writing, revising and proof-reading the manuscript. All authors read and approved the final manuscript.

### Conflict of interest statement

The authors declare that the research was conducted in the absence of any commercial or financial relationships that could be construed as a potential conflict of interest.
