# Supplementary material for: System Pharmacology-Based Dissection of the Synergistic Mechanism of Huangqi and Huanglian for Diabetes Mellitus
Source: Front Pharmacol. 2017 Oct 5;8:694. doi: 10.3389/fphar.2017.00694 (PMC5633780; doi:10.3389/fphar.2017.00694)
Supplement: Supplementary file 1 [file Presentation1.pdf]

Supplementary Material

System pharmacology-based dissection of the synergistic mechanism of Huangqi and Huanglian for diabetes mellitus

Shi-Jun Yue[1,2,3], Juan Liu[1,4], Wu-Wen Feng[4], Fei-Long Zhang[5], Jian-Xin Chen[5], Lan-Ting Xin[2,3], Cheng Peng[4], Hua-Shi Guan[2,3], Chang-Yun Wang[2,3*], Dan Yan[1*]

[*]**Corresponding authors:**

E-mail address: yd277@126.com (D. Yan) or changyun@ouc.edu.cn (C.-Y. Wang)

1 Supplementary Tables and Figures

Supplementary Table S1. The detailed information of antidiabetic drugs from DrugBank.

ID	Drug	CAS	MW	CLogP	nHDon	nHAcc
D1	Acarbose	56180-94-0	645.61	-6.80	14	19
D2	Alogliptin	850649-61-5	339.39	0.66	1	5
D3	Atorvastatin	134523-00-5	558.64	5.70	4	5
D4	Benazepril	86541-75-5	424.49	3.30	2	5
D5	Bromocriptine	25614-03-3	654.59	3.50	3	6
D6	Candesartan	139481-59-7	440.45	6.10	2	7
D7	Canagliflozin	42133-18-0	444.52	3.09	4	5
D8	Chlorpropamide	94-20-2	276.74	2.27	2	3
D9	Dapagliflozin	461432-26-8	408.87	2.52	4	6
D10	Desmopressin	16679-58-6	1069.22	-4.20	14	15
D11	Empagliflozin	864070-44-0	450.91	1.79	4	7
D12	Fosinopril	98048-97-6	563.66	6.30	1	5
D13	Gliclazide	21187-98-4	323.41	2.60	2	4
D14	Glyburide	10238-21-8	494.00	4.70	3	5
D15	Hydrochlorothiazide	58-93-5	297.74	-0.07	3	5
D16	Idelalisib	870281-82-6	415.43	3.03	2	6
D17	Linagliptin	668270-12-0	472.54	2.62	1	7
D18	Lisinopril	76547-98-3	405.49	-1.01	4	7
D19	Losartan	114798-26-4	422.91	6.10	2	5
D20	Metformin	657-24-9	129.16	12.40	4	5
D21	Miglitol	72432-03-2	207.22	-2.70	5	6
D22	Nateglinide	105816-04-4	317.42	2.40	2	3
D23	Olmesartan	144689-24-7	446.50	5.90	3	7
D24	Pioglitazone	111025-46-8	356.44	2.30	1	4
D25	Repaglinide	135062-02-1	452.59	5.90	2	5
D26	Rosiglitazone	122320-73-4	357.43	2.40	1	5
D27	Saxagliptin	361442-04-8	315.41	0.88	2	4
D28	Sitagliptin	486460-32-6	407.31	1.50	1	4
D29	Simvastatin	79902-63-9	418.57	4.68	1	3
D30	Tolazamide	1156-19-0	311.40	2.69	2	4
D31	Tolbutamide	64-77-7	270.35	2.34	2	3
D32	Trandolapril	87679-37-6	430.54	3.50	2	5
D33	Valsartan	137862-53-4	435.52	5.80	2	6
D34	Vildagliptin	274901-16-5	303.40	1.12	2	4

MW: molecular weight, nHDon: number of donor atoms for H-bonds, nHAcc: number of acceptor atoms for H-bonds and CLogP: Calculated octanol-water partition coeff.(logP).

Supplementary Table S2. The detailed information of all compounds in Huangqi and Huanglian.

ID	Herb	Compound	CAS	MW	CLogP	nHDon	nHAcc	OB	DL
M1	*Coptis chinensis*	Berberine	2086-83-1	336.39	3.45	0	4	0.68	0.78
M2	*Coptis chinensis*	Columbamine	3621-36-1	338.41	3.40	1	4	26.94	0.59
M3	*Coptis chinensis*	Berberrubine	15401-69-1	322.36	3.20	1	4	35.74	0.73
M4	*Coptis chinensis*	8-Oxocoptisine	19716-61-1	335.31	3.18	0	6	46.83	0.89
M5	*Coptis chinensis*	Javanicin	476-45-9	508.52	-1.24	0	12	17.38	0.78
M6	*Coptis chinensis*	Obacunoic acid	751-29-1	472.58	2.07	2	8	20.69	0.79
M7	*Coptis chinensis*	Obamegine	479-37-8	594.76	6.72	2	8	2.55	0.11
M8	*Coptis chinensis*	Fagarine	524-15-2	229.25	2.35	0	4	72.23	0.15
M9	*Coptis chinensis*	2-Carboxymethyl-3-prenyl-2,3-epoxy-1,4-naphthoquinone	N/A	300.33	2.46	0	5	20.68	0.26
M10	*Coptis chinensis*	Magnoflorine	7224-58-0	342.45	3.12	2	4	22.60	0.55
M11	*Coptis chinensis*	Magnograndiolide	92618-98-9	266.37	1.18	2	4	63.71	0.19
M12	*Coptis chinensis*	Epiberberine	6873-09-2	336.39	3.45	0	4	43.09	0.78
M13	*Coptis chinensis*	Groenlandicine (Dehydrocheilanthifoline)	38691-95-1	322.36	3.20	1	4	28.42	0.72
M14	*Coptis chinensis*	Corydaldine	493-49-2	207.25	1.08	1	4	49.30	0.09
M15	*Coptis chinensis*	Noroxyhydrastinine	21796-14-5	191.20	0.88	1	4	38.89	0.10
M16	*Coptis chinensis*	Phellodendrine	6873-13-8	342.45	3.19	2	4	2.50	0.58
M17	*Coptis chinensis*	Limonin (Obaculactone)	1180-71-8	470.56	1.42	0	8	21.30	0.57
M18	*Coptis chinensis*	*(R)*-Canadine	522-97-4	339.42	3.40	0	5	55.37	0.77
M19	*Coptis chinensis*	Berlambine (Oxyberberine)	549-21-3	351.38	2.49	0	6	36.68	0.82
M20	*Coptis chinensis*	Jatrorrhizine	3621-38-3	338.41	3.40	1	4	19.65	0.59
M21	*Coptis chinensis*	Palmatine	3486-67-7	352.44	3.65	0	4	64.60	0.65
M22	*Coptis chinensis*	Palmidin A	17062-55-4	510.52	4.52	6	8	35.36	0.65
M23	*Coptis chinensis*	Coptisine	3486-66-6	320.34	3.25	0	4	7.21	0.86
M24	*Coptis chinensis*	Corchoroside A	508-76-9	534.71	1.25	4	9	19.60	0.69
M25	*Coptis chinensis*	Corchoroside A_qt	N/A	404.55	1.34	3	6	104.95	0.78
M26	*Coptis chinensis*	Worenine	38763-29-0	334.37	3.73	0	4	45.83	0.87
M27	*Coptis chinensis*	Obacunone	751-03-1	454.56	2.68	0	7	43.29	0.77
M28	*Coptis chinensis*	Ethyl caffeate	102-37-4	208.23	1.97	2	4	103.85	0.07
M29	*Coptis chinensis*	Zosimin (Columbianadin)	5058-13-9	328.39	3.96	0	5	14.82	0.36
M30	*Coptis chinensis*	6-*O*-*E*-Feruloylajugol	N/A	524.57	-0.84	6	12	26.13	0.85
M31	*Coptis chinensis*	6-*O*-*E*-Feruloylajugol_qt	N/A	362.41	0.90	3	7	15.46	0.43
M32	*Coptis chinensis*	Ferulic acid	537-98-4	194.20	1.62	2	4	39.56	0.06
M33	*Coptis chinensis*	Vanillic acid	121-34-6	168.16	1.15	2	4	35.47	0.04
M34	*Coptis chinensis*	Hydroxytyrosol	10597-60-1	154.18	1.01	3	3	57.57	0.03

M35	*Coptis chinensis*	*p*-Coumaric acid	501-98-4	164.17	1.64	2	3	43.29	0.04
M36	*Coptis chinensis*	Heriguard (Chlorogenic acid)	327-97-9	354.34	-0.42	6	9	11.93	0.33
M37	*Coptis chinensis*	Clemastanin B_qt	112747-98-5	360.44	2.46	3	6	5.53	0.38
M38	*Coptis chinensis*	Tetrandrine	518-34-3	622.82	7.22	0	8	26.64	0.10
M39	*Coptis chinensis*	Trihydroxybufosterocholanic acid	53939-26-7	464.76	4.51	4	5	16.78	0.84
M40	*Coptis chinensis*	2,5-Dihydroxybenzoic acid	490-79-9	154.13	0.90	3	4	29.33	0.04
M41	*Coptis chinensis*	Methyl protocatechuate	2150-43-8	168.16	1.15	2	4	38.19	0.04
M42	*Coptis chinensis*	Cinnamic acid, 3,4-dimethoxy-(8CI)	2316-26-9	208.23	1.87	1	4	63.86	0.07
M43	*Coptis chinensis*	Pycnamine	569-16-4	608.79	6.97	1	8	27.61	0.11
M44	*Coptis chinensis*	Danshensu	76822-21-4	198.19	0.71	4	5	36.91	0.06
M45	*Coptis chinensis*	Moupinamide	65646-26-6	313.38	2.86	3	5	86.71	0.26
M46	*Coptis chinensis*	Oxyacanthine	548-40-3	608.79	6.97	1	8	9.91	0.11
M47	*Coptis chinensis*	5,8-Dihydroxy-2-(2-phenylethyl)chromone (DPEC)	69809-24-1	282.31	3.38	2	4	28.36	0.24
M48	*Coptis chinensis*	Ethyl protocatechuate	3943-89-3	182.19	1.50	2	4	35.77	0.05
M49	*Coptis chinensis*	Isovanillin	621-59-0	152.16	1.31	1	3	31.01	0.03
M50	*Coptis chinensis*	3-(3′,4′-Dihydroxyphenyl)-(2*R*)-lactic acid	76822-21-4	198.17	–0.25	4	5	46.30	0.06
M51	*Coptis chinensis*	3-(3′,4′-Dihydroxyphenyl)-(2*R*)-lactic acid 4′-*O*-*β*-D-glucopyranoside	96552-85-1	360.31	–2.27	1	7	38.45	0.05
M52	*Coptis chinensis*	3′,4′-Dihydroxyphenyl alcohol 1-*O*-*β*-D-glucopyranoside	76873-99-9	316.31	–1.19	6	8	57.57	0.03
M53	*Coptis chinensis*	Gentisic acid 5-*O*-*β*-D-glucopyranoside	1820-89-9	316.26	–0.42	6	9	17.50	0.33
M54	*Coptis chinensis*	4-*O*-Feruloyl-D-quinic acid	2613-86-7	368.34	–0.36	5	9	16.46	0.43
M55	*Coptis chinensis*	5-*O*-Feruloyl-D-quinic acid	40242-06-6	368.34	–0.14	5	9	15.46	0.43
M56	*Coptis chinensis*	Protocatechuic aldehyde	139-85-5	138.13	1.06	2	3	38.35	0.03
M57	*Coptis chinensis*	Wogonin	10-29-7	284.28	2.59	2	5	30.68	0.23
M58	*Coptis chinensis*	(+)-Isolariciresinol	548-29-8	360.44	2.25	4	6	15.08	0.39
M59	*Coptis chinensis*	(+)-Pinoresinol	487-36-5	358.42	2.13	2	6	4.25	0.52
M60	*Coptis chinensis*/ *Astragalus membranaceus*	*β*-Sitosterol	64997-52-0	428.82	8.54	1	1	36.23	0.78
M61	*Astragalus membranaceus*	Hederagenin	465-99-6	414.79	8.08	1	1	36.91	0.75
M62	*Astragalus membranaceus*	Lupeol	545-47-1	426.80	7.40	1	1	12.12	0.78
M63	*Astragalus membranaceus*	Astrachrysoside A	132160-35-1	769.09	0.54	8	13	24.55	0.10
M64	*Astragalus membranaceus*	3-Hydroxy-2-picoline	1121-25-1	109.14	0.70	1	2	62.47	0.02
M65	*Astragalus membranaceus*	5-*O*-Methylvisammioside	84272-85-5	452.50	0.27	4	10	5.38	0.81
M66	*Astragalus membranaceus*	5'-Hydroxyisomuronulatol-2',5'-*di*-*O*-glucoside	N/A	642.67	-0.95	9	16	41.72	0.69
M67	*Astragalus membranaceus*	5'-Hydroxyisomuronulatol-2',5'-*di*-*O*-glucoside_qt	N/A	480.51	0.96	6	11	3.65	0.80
M68	*Astragalus membranaceus*	7,2'-Dihydroxy-3',4'-dimethoxyisoflavone-7-*O*-*β*-D-glucoside	113235-89-5	476.47	0.40	5	11	16.16	0.86

M69	*Astragalus membranaceus*	7,2'-Dihydroxy-3',4'-dimethoxyisoflavone	1037248-17-1	314.31	2.30	2	6	5.45	0.30
M70	*Astragalus membranaceus*	Acetylastragaloside I	84687-47-8	911.21	0.79	6	17	43.54	0.09
M71	*Astragalus membranaceus*	Acetylastragaloside I_qt	N/A	749.05	2.54	3	12	30.75	0.17
M72	*Astragalus membranaceus*	Isoliquiritigenin	961-29-5	256.27	2.90	3	4	85.32	0.15
M73	*Astragalus membranaceus*	13-Hydroxy-9,11-octadecadienoic acid	5204-88-6	296.50	5.29	2	3	35.60	0.17
M74	*Astragalus membranaceus*	D-Arabinose	147-81-9	150.15	-2.17	4	5	1.87	0.02
M75	*Astragalus membranaceus*	D-Galacturonic acid, homopolymer	11100-11-1	194.16	-2.47	5	7	29.75	0.04
M76	*Astragalus membranaceus*	D-Glucuronic acid	87246-82-0	194.16	-2.47	5	7	3.35	0.04
M77	*Astragalus membranaceus*	Isoferulic acid	537-73-5	194.20	1.62	2	4	50.83	0.06
M78	*Astragalus membranaceus*	L-Fucopyranose	2438-80-4	164.18	-1.80	4	5	42.51	0.03
M79	*Astragalus membranaceus*	Bifendate	73536-69-3	418.38	2.56	0	10	31.10	0.67
M80	*Astragalus membranaceus*	*γ*-Aminobutyric acid	70582-09-1	103.14	-0.62	3	3	24.09	0.01
M81	*Astragalus membranaceus*	Ferulic acid (cis)	1014-83-1	194.20	1.62	2	4	54.97	0.06
M82	*Astragalus membranaceus*	Daidzein	486-66-8	254.25	2.33	2	4	19.44	0.19
M83	*Astragalus membranaceus*	Hirsutrin (Isoquercitrin)	482-35-9	464.41	-0.59	8	12	1.86	0.77
M84	*Astragalus membranaceus*	(2*R*,3*R*,4*S*,5*S*)-2,3,4,5-Tetrahydroxyhexanal hydrate	10030-85-0	164.18	-1.62	4	5	50.50	0.04
M85	*Astragalus membranaceus*	Soyasaponin I	51330-27-9	943.26	0.97	11	18	2.06	0.15
M86	*Coptis chinensis*/ *Astragalus membranaceus*	Choline	62-49-7	104.20	-1.57	1	1	0.47	0.01
M87	*Astragalus membranaceus*	(+)-Syringaresinol	21453-69-0	418.48	2.10	2	8	3.29	0.72
M88	*Astragalus membranaceus*	cis-*p*-Coumarate	4501-31-9	164.17	1.64	2	3	45.98	0.04
M89	*Astragalus membranaceus*	Isoflavanone	4737-27-3	316.33	2.42	2	6	109.99	0.30
M90	*Astragalus membranaceus*	Docosanoate	112-85-6	340.66	9.11	1	2	15.69	0.26
M91	*Astragalus membranaceus*	Flavaxin	130609-39-1	376.41	0.23	5	10	18.18	0.50
M92	*Astragalus membranaceus*	Astragaloside I	84680-75-1	869.17	0.41	7	16	46.79	0.11
M93	*Astragalus membranaceus*	Astragaloside I_qt	N/A	707.01	2.16	4	11	12.34	0.20
M94	*Astragalus membranaceus*	Astragaloside II	84676-89-1	827.13	0.03	8	15	0.79	0.13
M95	*Astragalus membranaceus*	Astragaloside II_qt	N/A	664.97	1.78	5	10	11.55	0.25
M96	*Astragalus membranaceus*	Astragaloside III	84687-42-3	785.09	-0.35	9	14	31.83	0.10
M97	*Astragalus membranaceus*	Astragaloside III_qt	N/A	622.93	1.40	6	9	5.35	0.32
M98	*Astragalus membranaceus*	Astragaloside IV (Astramembrannin I)	84687-43-4	785.09	-0.35	9	14	2.20	0.15
M99	*Astragalus membranaceus*	Astragaloside IV_qt	N/A	622.93	1.40	6	9	7.07	0.32
M100	*Astragalus membranaceus*	Astragaloside V	84687-44-5	933.05	–2.31	13	20	22.50	0.14
M101	*Astragalus membranaceus*	Astragaloside VI	84687-45-6	931.08	–1.29	12	19	16.44	0.12
M102	*Astragalus membranaceus*	Astragaloside VII	84687-46-7	933.05	–2.31	13	20	18.38	0.09
M103	*Astragalus membranaceus*	Astragaloside VIII	119556-00-2	913.11	–2.11	10	17	20.60	0.10
M104	*Astragalus membranaceus*	Isoastragaloside I	84676-88-0	869.04	2.61	7	16	37.80	0.14
M105	*Astragalus membranaceus*	Isoastragaloside II	86764-11-6	827.02	1.69	8	15	46.06	0.13

M106	*Astragalus membranaceus*	Isoastragaloside IV	136033-55-1	827.02	1.69	8	15	22.40	0.15
M107	*Astragalus membranaceus*	Astrasieversianin XV	101843-83-8	901.22	-0.70	10	17	11.19	0.07
M108	*Astragalus membranaceus*	Astraisoflavanin (Mucronulatol-7-*O*-glucoside)	131749-60-5	464.51	1.22	5	10	18.37	0.86
M109	*Astragalus membranaceus*	Cycloastragenol	78574-94-4	490.73	4.19	4	5	25.70	0.10
M110	*Astragalus membranaceus*	Mucronulatol	20878-97-1	302.35	3.13	2	5	4.22	0.26
M111	*Astragalus membranaceus*	Vanillic acid	121-34-6	168.16	1.15	2	4	35.47	0.04
M112	*Astragalus membranaceus*	Linolenic acid	60-33-3	280.50	6.39	1	2	41.90	0.14
M113	*Astragalus membranaceus*	Betulinic acid (mairin)	472-15-1	456.78	6.52	2	3	55.38	0.78
M114	*Astragalus membranaceus*	Heriguard (Chlorogenic acid)	327-97-9	354.34	-0.42	6	9	11.93	0.33
M115	*Astragalus membranaceus*	Jaranol (Kumatakenin)	3301-49-3	314.31	2.09	2	6	50.83	0.29
M116	*Astragalus membranaceus*	Rhamnocitrin	569-92-6	300.28	2.02	3	6	12.90	0.27
M117	*Astragalus membranaceus*	Alexandrin	83-48-7	576.95	6.34	4	6	20.63	0.63
M118	*Astragalus membranaceus*	Isorhamnetin	480-19-3	316.28	1.76	4	7	49.60	0.31
M119	*Astragalus membranaceus*	3,9-*di*-*O*-Methylnissolin	73353-82-9	314.36	2.89	0	5	53.74	0.48
M120	*Astragalus membranaceus*	Calycosin (7,3'-Dihydroxy-4'-methoxyisoflavone)	20575-57-9	284.28	2.32	2	5	47.75	0.24
M121	*Astragalus membranaceus*	Calycosin 7-*O*-*β*-D-glucopyranoside	20633-67-4	446.44	0.41	5	10	10.05	0.81
M122	*Astragalus membranaceus*	7-*O*-Methylisomucronulatol	137217-83-5	316.38	3.38	1	5	74.69	0.30
M123	*Astragalus membranaceus*	9,10-Dimethoxypterocarpan-3-*O*-*β*-D-glucoside	94367-42-7	462.49	0.74	4	10	36.74	0.92
M124	*Astragalus membranaceus*	Astrapterocarpan (6*aR*,11*aR*-3-Hydroxy-9,10-dimethoxypterocarpan)	73340-41-7	300.33	2.64	1	5	64.26	0.42
M125	*Astragalus membranaceus*	Formononetin	485-72-3	268.28	2.58	1	4	69.67	0.21
M126	*Astragalus membranaceus*	Ononin (Formononetin-7-*O*-*β*-D-glucoside)	486-62-4	430.44	0.68	4	9	11.52	0.78
M127	*Astragalus membranaceus*	Rhamnocitrin-3-*O*-glucoside	41545-37-3	462.44	-0.07	6	11	2.87	0.76
M128	*Astragalus membranaceus*	Asernestioside A	123914-38-5	931.25	-1.21	11	18	11.07	0.03
M129	*Astragalus membranaceus*	Asernestioside A_qt	N/A	769.09	0.54	8	13	24.55	0.10
M130	*Astragalus membranaceus*	Asernestioside B	123914-39-6	973.29	-0.83	10	19	12.54	0.03
M131	*Astragalus membranaceus*	Asernestioside B_qt	N/A	811.13	0.92	7	14	14.03	0.09
M132	*Astragalus membranaceus*	Isomucronulatol	64474-51-7	302.35	3.13	2	5	67.67	0.26
M133	*Astragalus membranaceus*	Isomucronulatol-7,2'-*di*-*O*-glucosiole	137217-84-6	626.67	-0.68	8	15	49.28	0.62
M134	*Astragalus membranaceus*	Isomucronulatol-7,2'-*di*-*O*-glucosiole_qt	N/A	464.51	1.22	5	10	23.42	0.79
M135	*Astragalus membranaceus*	Lupenone	1617-70-5	424.78	7.36	0	1	11.66	0.78
M136	*Astragalus membranaceus*	1,7-Dihydroxy-3,9-dimethoxy pterocarpene	N/A	314.31	3.11	2	6	39.05	0.48
M137	*Astragalus membranaceus*	Betaine	107-43-7	117.17	-2.04	0	2	40.92	0.01
M138	*Astragalus membranaceus*	Coumarin	91-64-5	146.15	1.90	0	2	29.17	0.04
M139	*Astragalus membranaceus*	Linolenic acid	60-33-3	278.48	5.95	1	2	45.01	0.15
M140	*Astragalus membranaceus*	Folic acid	59-30-3	441.45	0.01	7	13	68.96	0.71
M141	*Coptis chinensis/*	Rutin	115888-40-9	610.57	-1.45	10	16	3.20	0.68

	Astragalus membranaceus								
M142	*Astragalus membranaceus*	5,7,4′-Trihydroxyisoflavone (Genistein)	446-72-0	270.25	2.07	3	5	17.93	0.21
M143	*Astragalus membranaceus*	α-D-Ribopyranose	7296-59-5	150.15	-2.17	4	5	51.08	0.02
M144	*Astragalus membranaceus*	Nicotinic acid	59-67-6	123.12	0.28	1	3	47.65	0.02
M145	*Astragalus membranaceus*	L-Arginine	142-49-4	174.24	-1.11	7	6	47.64	0.03
M146	*Astragalus membranaceus*	L-Prolinum	4305-67-3	115.15	-0.06	2	3	77.57	0.01
M147	*Astragalus membranaceus*	Palmitic acid	67701-02-4	256.48	6.37	1	2	19.3	0.10
M148	*Coptis chinensis*/ *Astragalus membranaceus*	Quercetin	117-39-5	302.25	1.50	5	7	46.43	0.28
M149	*Astragalus membranaceus*	Soyasaponin II	55319-36-3	913.23	1.48	10	17	2.10	0.05
M150	*Astragalus membranaceus*	Apigenin	520-36-5	270.25	2.33	3	5	23.06	0.21
M151	*Astragalus membranaceus*	Caffeic acid	331-39-5	180.17	1.37	3	4	54.97	0.05
M152	*Astragalus membranaceus*	Riboflavin	83-88-5	376.41	0.23	5	10	6.79	0.50
M153	*Astragalus membranaceus*	Soyasapogenol B	595-15-3	458.80	5.11	3	3	16.73	0.75
M154	*Coptis chinensis*/ *Astragalus membranaceus*	Kaempferol	520-18-3	286.25	1.77	4	6	41.88	0.24
M155	*Astragalus membranaceus*	β-Daucosterol	474-58-8	548.89	5.93	4	6	20.18	0.69
M156	*Astragalus membranaceus*	Lariciresinol	27003-73-2	360.44	2.46	3	6	5.53	0.38
M157	*Astragalus membranaceus*	Isoquercitrin_qt	491-54-3	300.28	2.02	3	6	73.41	0.27
M158	*Astragalus membranaceus*	L-3-Hydroxy-9-methoxypterocarpan	32383-76-9	270.30	2.66	1	4	49.22	0.34
M159	*Astragalus membranaceus*	Astramembrannin II	83207-60-7	664.88	3.62	5	10	7.07	0.32
M160	*Astragalus membranaceus*	L-Canavanine	543-38-4	176.21	-1.84	7	7	54.95	0.03
M161	*Astragalus membranaceus*	9,10-Dimethoxypterocarpan-7-*O*-β -D-glucopyranoside	438000-10-3	462.45	0.71	4	10	36.74	0.92
M162	*Astragalus membranaceus*	Astragalin	480-10-4	448.41	-0.32	7	11	14.03	0.74
M163	*Astragalus membranaceus*	Acetytastragaloside-I	84687-47-8	911.21	0.79	6	17	43.54	0.09
M164	*Astragalus membranaceus*	Astrasieversianin X	101858-42-8	754.96	1.63	8	13	11.19	0.07
M165	*Astragalus membranaceus*	Astrojanoside A	223924-16-1	1076.26	–0.73	14	22	1.86	0.02
M166	*Astragalus membranaceus*	Azukisaponin V	82793-05-3	458.80	5.11	3	3	16.99	0.79
M167	*Astragalus membranaceus*	Cyclocephaloside I	205312-83-0	784.98	1.21	9	14	22.50	0.10
M168	*Astragalus membranaceus*	Malonylastragaloside	1254039-91-2	955.10	2.10	7	19	2.07	0.05
M169	*Astragalus membranaceus*	L-Asparagine	328-41-6	132.14	-1.85	5	5	83.96	0.02
M170	*Astragalus membranaceus*	Folinic acid	58-05-9	473.50	-0.04	8	14	23.60	0.74
M171	*Astragalus membranaceus*	Astragalus polysaccharides	N/A	N/A	N/A	N/A	N/A	N/A	N/A

OB: oral bioavailability, DL: druglikeness, MW: molecular weight, nHDon: number of donor atoms for H-bonds, nHAcc: number of acceptor atoms for H-bonds and CLogP: Calculated octanol-water partition coeff.(logP).

qt represents the molecule with deglycosylation.

Supplementary Table S3. The detailed docking information of active ingredients from Huangqi and Huanglian and their potential targets by Surflex-Dock.

Proname	Molname	Total_Score	Crash	Polar	D_Score	PMF_Score	G_Score	ChemScore	Unified_CScore	Global_CScore
GLUT2	Rhamnocitrin-3-O-glucoside	-71.8079	-82.9294	3.0228	-230.272	67.9523	-434.249	-37.5791	4	4
GLUT2	Kaempferol	-71.2324	-82.0636	2.3157	-229.643	67.2966	-419.95	-37.434	4	4
GLUT2	Berberrubine	-70.1674	-79.0858	3.3483	-362.703	122.6626	-286.556	-40.6663	4	4
GLUT2	Ferulic acid	-62.5278	-68.8373	0.0011	-288.776	199.7404	-323.101	-57.4606	3	3
GLUT2	Obacunone	-61.0381	-67.4493	0.7471	-349.85	100.927	-405.561	-43.7517	4	4
GLUT2	Berlambine	-54.1282	-62.1562	0.0098	-289.025	221.2907	-336.337	-58.4355	3	3
GLUT2	Columbamine	-53.4067	-57.3971	2.1586	-317.98	305.6717	-293.058	-47.5078	4	4
GLUT2	(*R*)-Canadine	-52.6808	-59.5689	0.0001	-281.849	196.813	-282.974	-58.8931	3	3
GLUT2	Magnoflorine	-48.0442	-52.988	2.1291	-329.191	62.5245	-334.467	-42.6956	4	4
GLUT2	Phellodendrine	-42.276	-48.3702	0.0204	-279.844	210.4894	-309.928	-59.1452	3	3
GLUT2	9,10-Dimethoxypterocarpan-3-O-β-D-glucoside	-38.0406	-44.4775	0.0001	-157.673	18.7584	-241.973	-25.6806	4	4
GLUT2	7-O-methylisomucronulatol	-37.3009	-42.7216	0	-146.902	19.4536	-224.928	-24.653	4	4
GLUT2	Berberine	-36.5373	-41.9437	2.8671	-323.192	292.1928	-314.291	-47.0892	4	4
GLUT2	Lupeol	-25.8266	-32.9892	0.2929	-193.247	-36.2966	-353.842	-27.3278	5	5
GLUT2	Astragaloside III	-25.3446	-31.711	0.0287	-159.423	-1.8998	-265.302	-31.9126	5	5
GLP1R	Lupeol	-24.2898	-31.2068	0.0089	-174.886	-23.4066	-292.233	-22.7787	4	4
GLUT2	Calycosin	-24.1911	-31.3782	3.3648	-177.707	-14.6625	-245.98	-23.9781	5	5
GLP1R	Isorhamnetin	-24.1246	-30.903	0.0755	-178.772	-22.6816	-302.086	-22.8746	4	4
GLUT2	Astragaloside IV	-24.1061	-34.1111	1.8831	-166.474	16.1648	-302.281	-27.2481	5	5
GLP1R	Hederagenin	-23.5866	-33.7756	1.281	-192.533	5.2851	-337.866	-32.7915	4	4
GLUT2	Astragaloside II	-23.3374	-30.7031	0.6959	-154.857	8.4101	-290.504	-27.1846	5	5
GLUT2	Astragaloside I	-22.945	-30.0352	0.0179	-168.374	2.7633	-276.399	-32.7495	5	5
GLP1R	Epiberberine	-20.953	-26.9593	0.4349	-119.667	-30.6768	-219.615	-19.7004	5	5
IDE	Vanillic acid	-20.8176	-28.1625	0.0865	-179.681	-88.5524	-278.44	-22.5528	4	4
IDE	Jatrorrizine	-20.0095	-27.1335	0.7333	-179.854	-104.3024	-274.307	-22.5939	4	4
GLP1R	Ferulic acid	-18.3878	-24.3402	0.9783	-118.178	-50.0936	-243.564	-23.5984	3	3
GLP1R	Berlambine	-16.0235	-20.8294	1.045	-111.863	-47.7554	-237.04	-24.7655	3	3
GLUT2	Isorhamnetin	-5.2172	-12.1222	0.5711	-134.278	-25.3184	-217.608	-18.3729	4	4
GLUT2	Hederagenin	-4.3543	-11.3753	0	-136.572	-21.4423	-226.331	-18.5703	4	4
GLUT2	Palmatine	-4.3239	-11.2835	2.1095	-158.239	-67.2785	-213.421	-27.6681	5	5
GLUT2	Groenlandicine	-4.1843	-12.4857	0.1434	-175.952	-51.8414	-309.515	-28.8203	3	3
GLUT2	Rutin	-4.0273	-9.994	2.5205	-136.079	-66.3032	-186.933	-21.9948	4	4
GLUT2	Epiberberine	-3.9709	-9.8833	0	-104.936	5.6398	-222.578	-15.9405	4	4

GLP1R	Berberrubine	-3.8172	-10.8185	0.2279	-109.526	-17.2602	-261.528	-21.6737	4	4
GLP1R	Obacunone	-3.4179	-11.0416	0.0023	-110.246	-4.3134	-248.286	-20.1544	4	4
HNF1A	Obacunone	-3.3831	-9.8443	0.0021	-162.848	23.2717	-291.95	-25.8559	3	3
GLUT2	Vanillic acid	-3.1077	-11.8177	0	-134.533	-16.9053	-241.635	-19.3521	4	4
GLUT2	Jatrorrizine	-2.8245	-11.0202	0	-147.68	-10.8298	-267.558	-20.5669	4	4
GLP1R	Columbamine	-1.9168	-7.3639	0	-122.427	-13.3716	-260.308	-18.3257	4	4
GLP1R	Magnoflorine	-1.8778	-9.1275	0	-103.5	-31.6935	-242.789	-18.834	4	4
GLP1R	Berberine	-1.7792	-7.8192	0	-121.247	-7.6952	-278.755	-17.9923	4	4
GLUT2	3,9-di-O-Methylnissolin	-1.0679	-9.4569	2.3303	-116.911	-31.3969	-258.448	-25.4068	5	5
HNF1A	Magnoflorine	-0.0787	-7.4807	0.9285	-131.25	4.807	-238.619	-22.876	3	3

Supplementary Table S4. The number of targets and references, network based efficacy (NE), and contribution indexes (CI) of active ingredients in Huangqi and Huanglian.

No.	No. targets	NE	No. references	CI	No.	No. targets	NE	No. references	CI
M1	33	149	219	76.40	M94	4	17	1	0.04
M2	17	72	2	0.34	M96	8	32	0	0
M3	17	68	6	0.96	M98	21	88	14	2.88
M4	12	52	0	0	M104	2	8	1	0.02
M10	20	79	6	1.11	M109	10	30	0	0
M12	21	75	9	1.58	M115	9	29	0	0
M13	14	53	2	0.25	M118	19	51	3	0.36
M16	16	81	0	0	M119	2	4	0	0
M18	21	88	0	0	M120	21	71	6	1.00
M19	14	55	0	0	M121	3	5	1	0.01
M20	16	65	8	1.22	M122	4	28	0	0
M21	19	71	11	1.83	M123	9	27	0	0
M23	16	52	14	1.70	M124	4	10	0	0
M26	11	42	0	0	M125	19	70	5	0.82
M27	4	11	1	0.03	M126	9	23	0	0
M32	8	23	14	0.75	M127	12	62	0	0
M33	4	14	6	0.20	M132	3	6	0	0
M60	10	24	12	0.67	M141	11	25	16	0.94
M61	11	20	0	0	M148	18	44	21	2.16
M62	22	85	8	1.59	M154	16	40	15	1.40
M85	1	2	0	0	M171	6	24	31	1.74
M92	7	25	0	0					

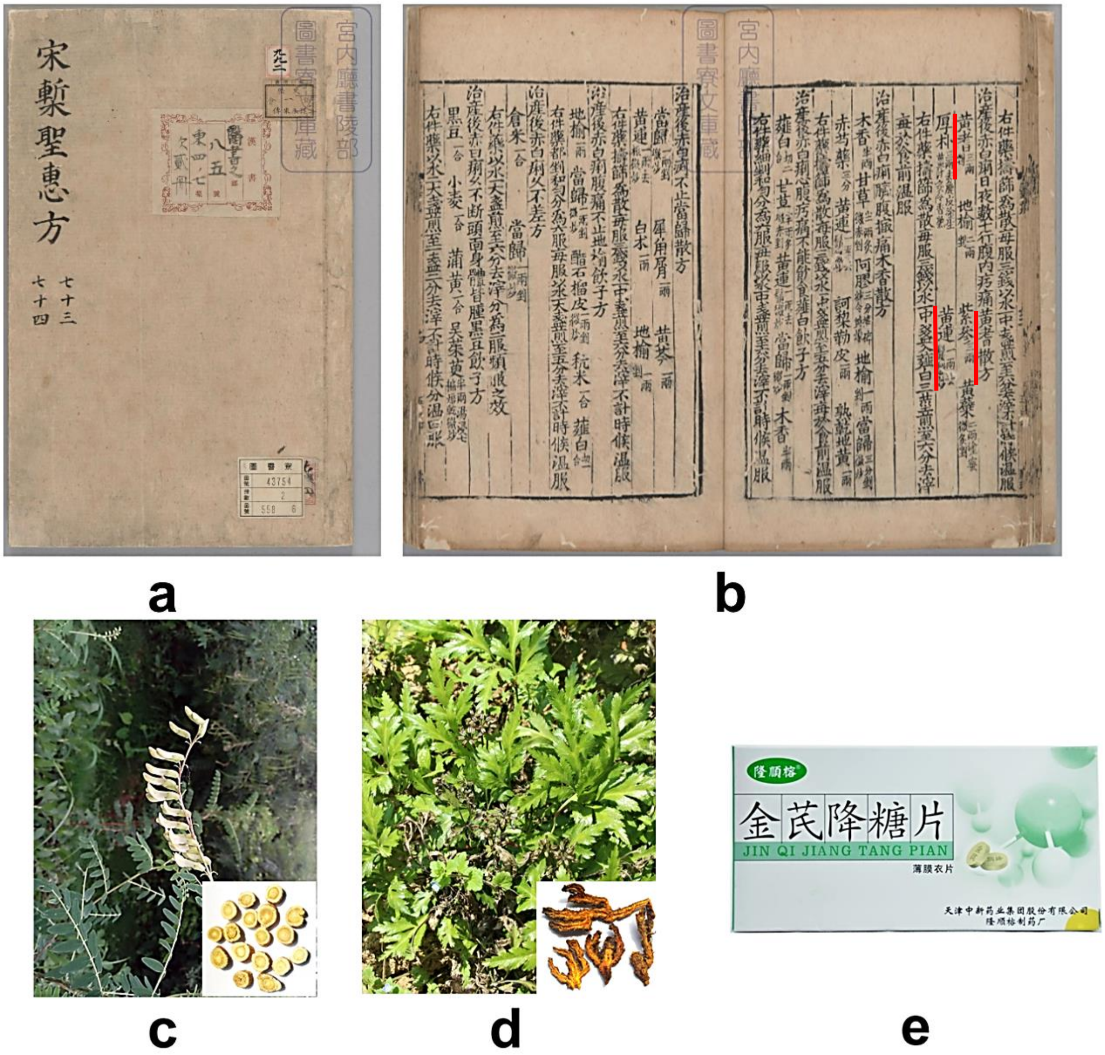

Supplementary Figure S1. The detailed information about Huangqi and Huanglian and their combination in TCM. **a** and **b** (download from http://db.sido.keio.ac.jp/kanseki/T_bib_search.php) refer to Huangqi San in *Taiping Shenhui Fang*; **c** and **d** (photographed by Hui-Wei Li and Shi-Jun Yue) refer to the plant and herbal pieces of Huangqi and Huanglian, respectively; additionally, **e** (photographed by Shi-Jun Yue) is the packing box of Jinqi Jiangtang tablets.

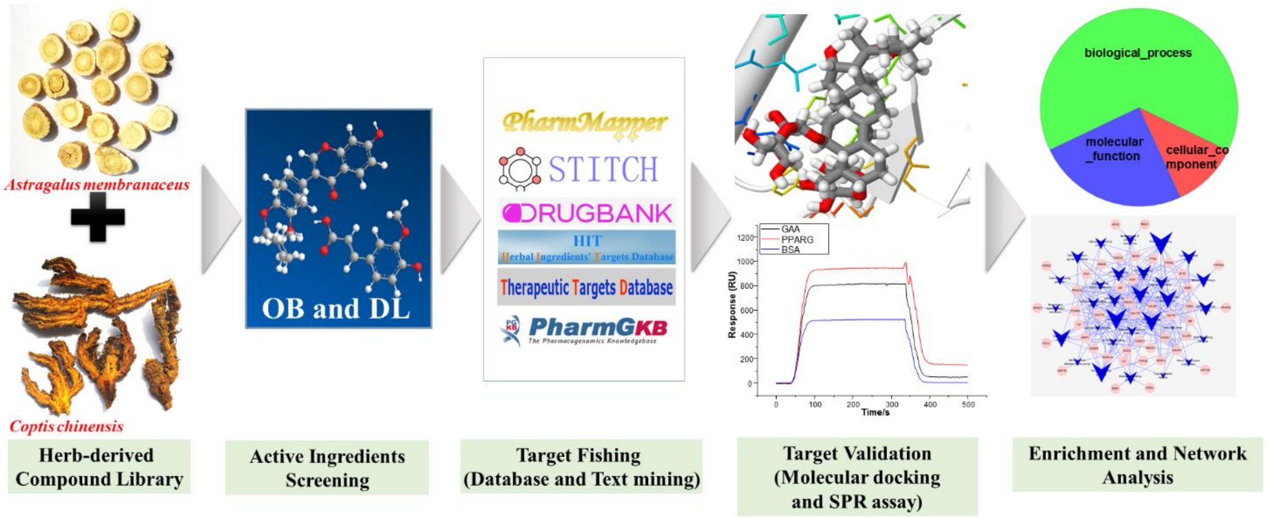

Supplementary Figure S2. The whole framework based on an integration strategy of system pharmacology.

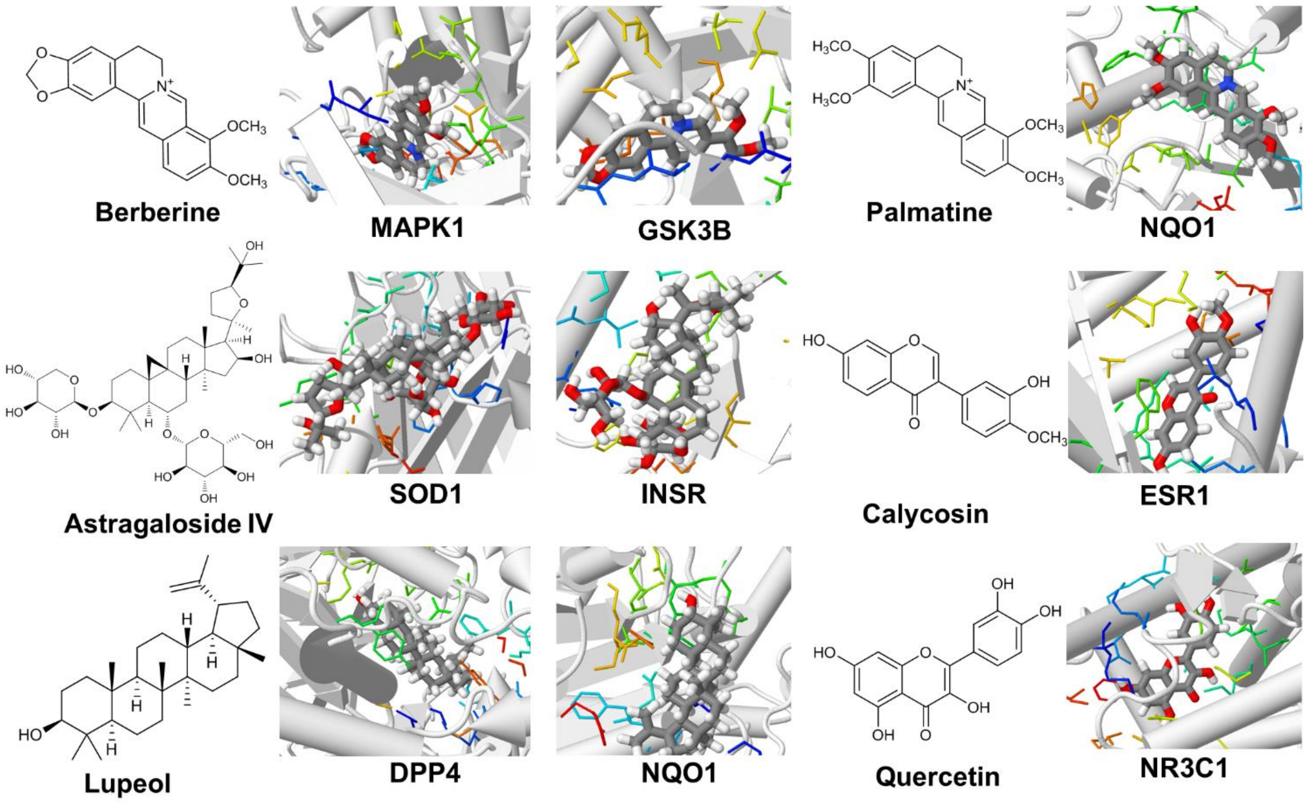

Supplementary Figure S3. Molecular docking analysis for active ingredients of Huangqi and Huanglian with their putative targets. The figures are produced by DRAR-CPI sever (Luo, et al., 2011).

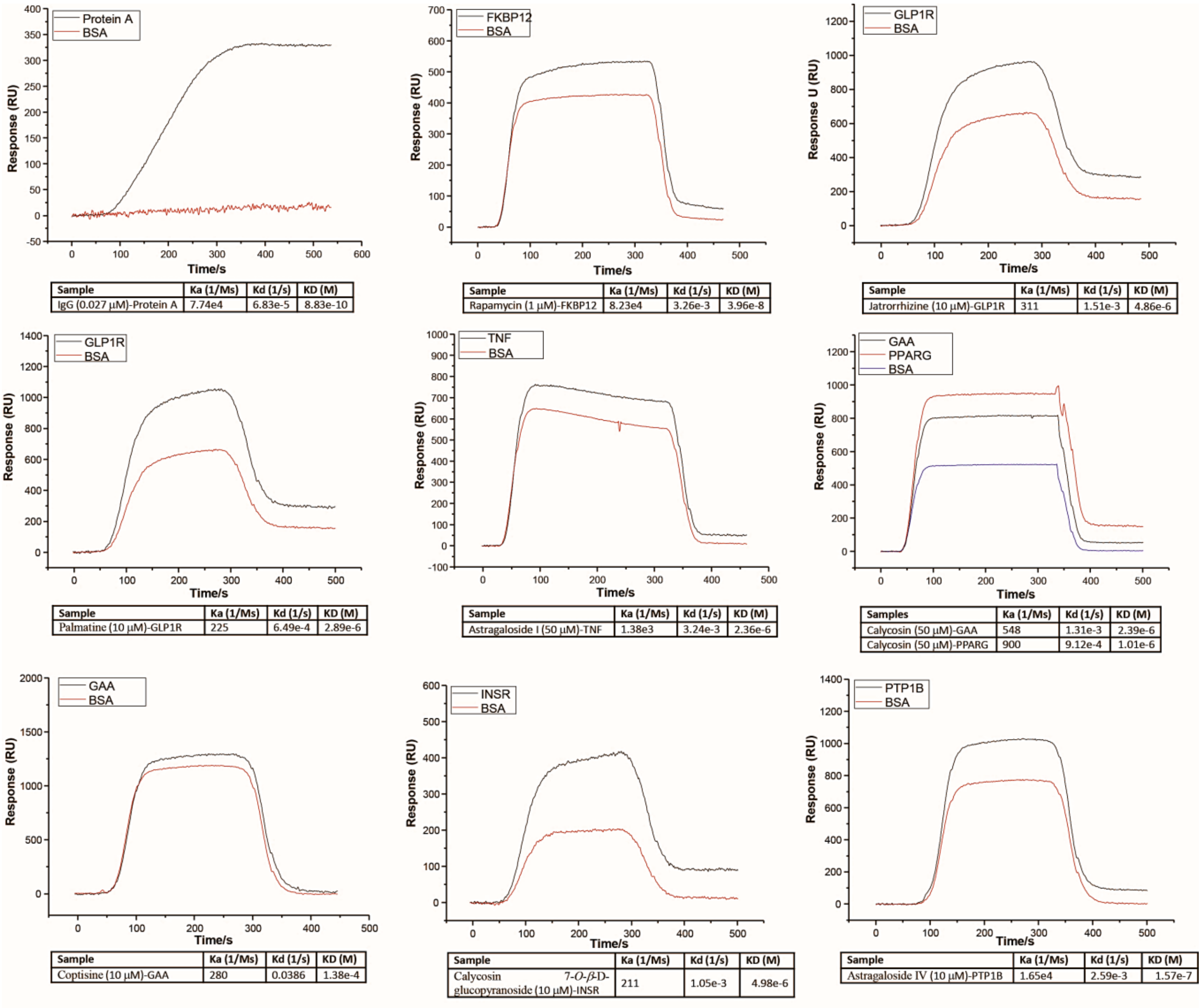

Sample	Ka (1/Ms)	Kd (1/s)	KD (M)
IgG (0.027 μM)-Protein A	7.74e4	6.83e-5	8.83e-10

Sample	Ka (1/Ms)	Kd (1/s)	KD (M)
Rapamycin (1 μM)-FKBP12	8.23e4	3.26e-3	3.96e-8

Sample	Ka (1/Ms)	Kd (1/s)	KD (M)
Jatrorrhizine (10 μM)-GLP1R	311	1.51e-3	4.86e-6

Sample	Ka (1/Ms)	Kd (1/s)	KD (M)
Palmatine (10 μM)-GLP1R	225	6.49e-4	2.89e-6

Sample	Ka (1/Ms)	Kd (1/s)	KD (M)
Astragaloside I (50 μM)-TNF	1.38e3	3.24e-3	2.36e-6

Samples	Ka (1/Ms)	Kd (1/s)	KD (M)
Calycosin (50 μM)-GAA	548	1.31e-3	2.39e-6
Calycosin (50 μM)-PPARG	900	9.12e-4	1.01e-6

Sample	Ka (1/Ms)	Kd (1/s)	KD (M)
Coptisine (10 μM)-GAA	280	0.0386	1.38e-4

Sample	Ka (1/Ms)	Kd (1/s)	KD (M)
Calycosin 7-*O*-*β*-D-glucopyranoside (10 μM)-INSR	211	1.05e-3	4.98e-6

Sample	Ka (1/Ms)	Kd (1/s)	KD (M)
Astragaloside IV (10 μM)-PTP1B	1.65e4	2.59e-3	1.57e-7

Supplementary Figure S4. Kinetic analysis of the binding of the active compounds in Huangqi and Huanglian to their putative targets using the PlexArray®HT instrument. Interaction of Rapamycin with FKBP12 and IgG with Protein A as a positive control.

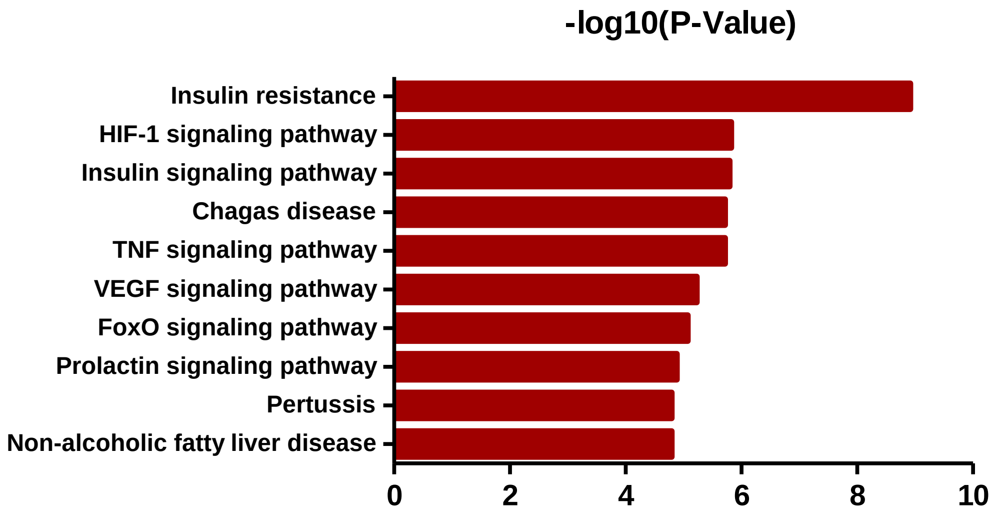

Supplementary Figure S5. Enriched KEGG pathways of potential targets of Huangqi and Huanglian.

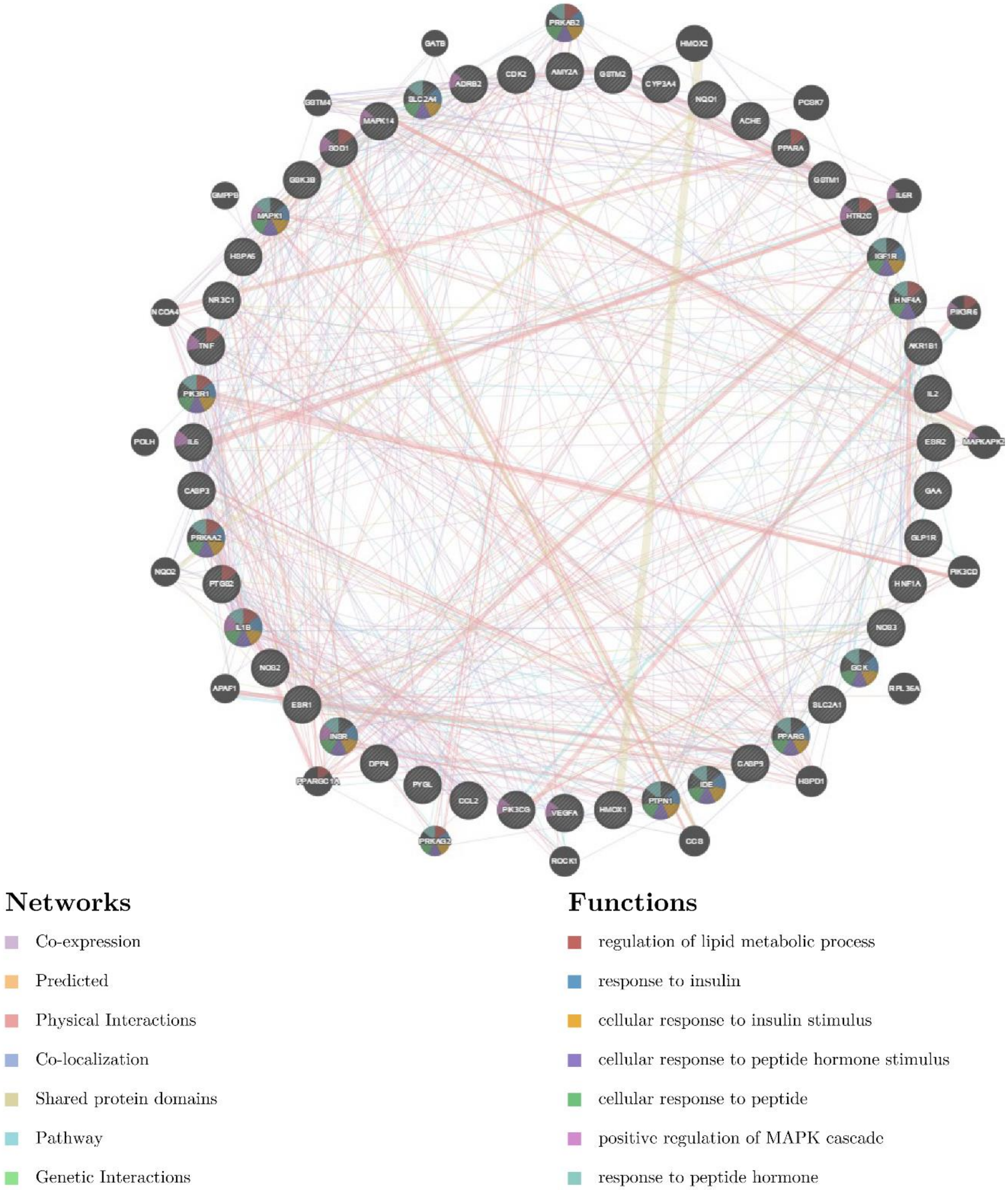

Supplementary Figure S6. Network and functional association of potential targets of Huangqi and Huanglian using GeneMANIA.

2 Supplementary Methods

2.1 Molecular docking

3D structures of active ingredients in Huangqi and Huanglian were obtained from the PubChem Compound Database (http://www.ncbi.nlm.nih.gov/pccompound). Also, 3D structures of human proteins related to diabetes were obtained from protein data bank (PDB, www.rcsb.org/pdb/home/home.do). During downloading 3D structures of human proteins, we filtered the small molecules by three criterias: (1) the 3D structures without mutation and missing residues around the active site; (2) the 3D structures with high resolution; (3) for the selected protein structures with 2 or more chains, the single chains around the active sites were reserved for docking.

As one of the superior tools available for accurate docking, surflex flexible molecular docking method combines Hammerhead's empirical scoring function with a molecular similarity method to generate putative poses of ligand fragments. The interactions of human proteins and active ingredients were evaluated by Surflex-Dock search algorithm (Jain, A. N., 2003), which were further ranked by Global scores and Total scores. The binding proteins were finally selected as the human targets of active ingredients in Huangqi and Huanglian as their Total score < 0.

2.2 Binding affinity measurements

Direct affinity measurements for binding of the small molecules to TNF, GAA and PPARG were performed using the Plexera PlexArray HT system (Plexera LLC, Woodinville, WA, USA) as described previously (Singh, et al., 2015). Briefly, proteins were immobilized to the surface of a biochip Graft-to-then-from-COOH via PDMS using the SpotBot3 Microarrayer system. Small molecules (20 μM or 50 μM in PBST buffer) was used and the interactions between the proteins with different small molecules were determined by SPR imaging using the Plexera PlexArray HT system. The binding data were analyzed using the PLEXERA SPR Date Analysis Module. k_a is the association rate constant for the binding, k_d is the dissociation rate constant for the complex.

Affinities were calculated, in the kinetic analysis, from the relation $K_D = k_d / k_a$ in the equilibrium analysis.

2.3 Analysis by GeneMANIA

GeneMANIA was proposed for predicting gene functions in real time, and it is composed of a heuristic algorithm derived from a ridge regression to integrate multiple networks through a process of label propagation (Warde-Farley, et al., 2010). Gene function prediction website (GeneMANIA: http://www.genemania.org/) was used for correlation analysis between genes.

References

Jain, A. N. (2003). Surflex: fully automatic flexible molecular docking using a molecular similarity-based search engine. *J. Med. Chem.* **46**, 499-511.

Luo, H., Chen, J., Shi, L.M., Mikailov, M., Zhu, H., Wang, K.J., He, L., Yang, L. (2011). DRAR-CPI: a server for identifying drug repositioning potential and adverse drug reactions via the chemical-protein interactome. *Nucleic Acids Res.* **39**, W492–498.

Singh, V., Singh, K., Nand, A., Dai, H. Q., Wang, J. G., Zhang, L. X., Merino, A., Zhu, J. S. (2015). Small molecule microarray screening methodology based on surface plasmon resonance imaging. *Arab. J. Chem.* http://dx.doi.org/10.1016/j.arabjc.2014.12.020.

Warde-Farley, D., Donaldson, S. L., Comes, O., Zuberi, K., Badrawi, R., Chao, P., Franz, M., Grouios, C., Kazi, F., Lopes, C. T., Maitland, A., Mostafavi, S., Montojo, J., Shao, Q., Wright, G., Bader, G. D., Morris, Q. (2010). The GeneMANIA prediction server: biological network integration for gene prioritization and predicting gene function. *Nucleic Acids Res.***38**, W214–W220.
